# Comparative Analysis of Glucagon Receptor Agonists vs. Resmetirom in MASLD and MASH: Network Meta‐Analysis of Clinical Trials

**DOI:** 10.1002/edm2.70157

**Published:** 2025-12-30

**Authors:** Celina R. Andonie, Alaaeddin Abusalameh, Ibrahim Ismail, Tamer Hodrob, Mahmoud Ladadweh, Hazem Ayesh

**Affiliations:** ^1^ Faculty of Medicine Al‐Quds University Jerusalem Palestinian Territory; ^2^ Deaconess Health System Evansville Indiana USA

## Abstract

**Background:**

MASLD and its progressive form MASH represent a global public health challenge due to their rising prevalence and their possible progression to cirrhosis and HCC. Resmetirom, dual (e.g., cotadutide, survodutide), and triple GRAs (e.g., retarutide) have demonstrated potential efficacy in recent clinical trials. This network meta‐analysis evaluates the comparative efficacy and safety of these treatments.

**Methods:**

We systematically searched PubMed, Scopus, ClinicalTrials.gov, and Cochrane Central for randomised controlled trials evaluating these medications versus placebo in adults with MASLD and MASH. The outcomes assessed included changes in ALT, AST, LDL, and HDL levels, changes in MRI‐PDFF, safety outcomes (diarrhoea, fatigue, and nausea), serious adverse events, ELF, adiponectin, and MASH resolution with no worsening of fibrosis. Random‐effects model and network meta‐analysis methods were employed.

**Results:**

Six trials met the inclusion criteria. GRAs significantly reduced ALT levels with MD of −22.10, while resmetirom demonstrated the greatest reduction in AST levels with a MD of −13.17. Resmetirom also led to a borderline significant increase in HDL with the most significant reduction in LDL levels. Moreover, GRAs showed a significant effect on MRI‐PDFF with a MD of −46.09. Overall, resmetirom showed a more favourable safety profile. In addition, GRAs significantly decreased ELF scores, resmetirom significantly improved MASH resolution without worsening of fibrosis, and both treatments significantly increased adiponectin.

**Conclusion:**

GRAs superiorly reduce ALT levels, MRI‐PDFF, and ELF. Resmetirom significantly reduces AST, HDL, and LDL levels, increases MASH resolution without worsening of fibrosis, and offers a more favourable safety profile. Both GRAs and resmetirom significantly increase adiponectin.

## Graphical abstract

This network meta‐analysis and systematic review builds on existing randomised controlled trials to compare the efficacy and safety of novel therapeutic agents like GRAs in comparison with resmetirom for the treatment of MASLD and MASH.

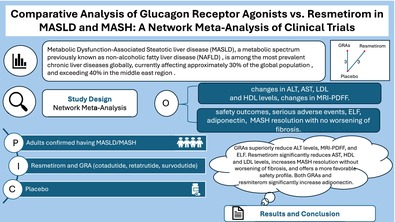

## 1 Introduction

Metabolic Dysfunction‐Associated Steatotic liver disease (MASLD), a metabolic spectrum previously known as non‐alcoholic fatty liver disease (NAFLD) [1], is among the most prevalent chronic liver diseases globally, currently affecting approximately 30% of the global population [2], and exceeding 40% in the middle east region [3]. The prevalence of MASLD in the United States is expected to exceed 100 million cases by 2030. It is also predicted to become the leading indication for liver transplantation, making it a growing public and serious health concern [4]. Approximately 16% of individuals with MASLD were identified as having an inflammatory Metabolic Dysfunction‐Associated Steatohepatitis (MASH), previously known as NASH, with global prevalence estimated at approximately 5% [2].

MASLD is closely linked with obesity, type 2 diabetes, and insulin resistance, and can progress to MASH, cirrhosis, and hepatocellular carcinoma. Both, MASLD and MASH place a significant economic burden on the U.S. healthcare system, with direct medical costs reaching billions of dollars annually. Moreover, patients with these conditions often require more frequent hospitalizations and outpatient care, which adds further pressure on the healthcare resources [5]. Despite its rising prevalence, there are still few FDA‐approved pharmacological treatments for MASLD and MASH. While our understanding of the underlying disease pathophysiology has improved significantly over the years, relatively slow progress has still been made in finding a definitive treatment, even after all of the intense research that has taken place over these years [6].

Despite the fact that lifestyle modification remains the cornerstone of MASLD and MASH management, several pharmacologic agents have shown promising results, particularly with the conditional FDA approval of resmetirom in 2024 [2] as the first disease specific treatment for MASLD [7]. Resmetirom is a thyroid hormone receptor‐β agonist, which has shown promising results in improving liver enzyme levels and reducing liver fat [8, 9, 10]. Additional range of promising pharmacological agents is currently under clinical investigation, including glucagon‐like‐peptide‐1 receptor agonists (GLP‐1RAs), sodium‐glucose cotransported‐2 (SGLT‐2) inhibitors, and fibroblast growth factor (FGF) analogues, all of which target specific pathophysiological mechanisms related to disease progression [11].

GLP‐1RAs have demonstrated promising results in the management of MASH and MASLD, particularly through their beneficial effect on key metabolic dysfunction including improvement in insulin sensitivity, weight reduction, and improvement in hepatic steatosis [12]. In a Randomised Clinical Trial (RCT), semaglutide administered at a daily dose of 0.4 mg led to MASH resolution without worsening of fibrosis in 59% of patients versus 17% with placebo [13]. Similarly, liraglutide was associated with histological resolution of MASH in 39% of treated patients, compared to 9% in the placebo group, without evidence of fibrosis progression [14].

In addition to GLP‐1RAs, new dual and triple agonists are being developed to simultaneously target multiple metabolic pathways. These agents have provided superior efficacy in reducing hepatic steatosis and improving overall metabolic control. Notable examples include the dual GLP‐1/glucagon receptor agonists cotadutide and survodutide, as well as the triple agonist retatrutide, which targets GLP‐1, GIP, and glucagon receptors [11]. However, individual RCTs have reported variable outcomes, and no single agent has emerged as the preferred treatment. A previous meta‐analysis was done to compare resmetirom with GLP‐1RAs [5]. But direct head‐to‐head trials comparing GRAs with resmetirom are limited, leading to uncertainty in clinical decision‐making. We used network meta‐analysis to integrate both direct and indirect evidence across multiple interventions (See Figure S2). This approach facilitated treatment ranking to provide clinicians with a clearer understanding of how GRAs and resmetirom compare within the broader therapeutic landscape.

This network meta‐analysis aims to evaluate the efficacy and safety of novel therapeutic agents like GRAs in comparison with resmetirom for the treatment of MASLD and MASH. Despite the many clinical trials that have been conducted, there is still a lack of thorough comparative analyses that provide guidelines for clinical practice. Our meta‐analysis builds on existing RCTs studies and focuses on key outcomes such as alanine aminotransferase (ALT) and aspartate aminotransferase (AST) levels, low density lipoprotein (LDL) and high density lipoprotein (HDL) levels, liver fat reduction (MRI‐PDFF), adverse events, serious adverse events, enhanced liver fibrosis (ELF), adiponectin, and MASH resolution with no worsening of fibrosis to offer a comparative perspective to shape future treatment options. The analysis includes only RCTs and synthesises direct and indirect evidence through a frequentist NMA framework.

## 2 Methods

### 2.1 Protocol and Reporting Standards

This systematic review and network meta‐analysis was conducted in accordance with the Preferred Reporting Items for Systematic Reviews and Meta‐Analyses (PRISMA) guidelines [15]. The PRISMA checklist was followed throughout the study (See Table S11). The protocol was prospectively registered on the Open Science Framework (OSF; Registration DOI: 10.17605/OSF.IO/E4J5F) [16]. The study design incorporated a frequentist framework using the netmeta R package to synthesize direct and indirect evidence from RCTs [17].

### 2.2 Eligibility Criteria and Study Selection

We included RCTs assessing pharmacologic therapies in adult patients with MASLD and MASH. Eligible interventions included dual GRAs (e.g., cotadutide, survodutide), triple GRAs (e.g., retarutide), and resmetirom. The comparator was a placebo. Studies were required to report at least one of the following outcomes: changes in liver enzymes (ALT/AST), changes in LDL/HDL levels, hepatic fat content (MRI‐PDFF), adverse events, serious adverse events, ELF, adiponectin, or MASH resolution with no worsening of fibrosis (See Table 1). Trials that focused exclusively on paediatric populations, did not report the outcomes of interest, involved non‐pharmacologic interventions, were conference abstracts without full‐text availability, or were post hoc analyses and extension studies were excluded (See Table S3).

**TABLE 1:** Study characteristics and outcomes in the included clinical trials. The table summarizes various clinical trials, detailing study design, registration, duration, treatment arms, primary outcomes, and population characteristics.

Study	Design	Registration	Duration	Treatment arms	Primary outcomes	Population
Harrison, 2019	Phase 2, RCT	NCT02912260	36 weeks	Resmetirom (80 mg), Placebo	Relative change in MRI‐PDFF assessed hepatic fat compared with placebo	Biopsy‐confirmed MASH, MRI‐PDFF ≥ 9%–10%, adults ≥ 18 years
Harrison, 2024	Phase 3, RCT	NCT03900429	52 weeks	Resmetirom (80 mg, 100 mg) Placebo	MASH resolution/improvement in fibrosis by at least one stage with no worsening of the MASLD activity score	Biopsy‐confirmed MASH, adults ≥ 18 years
Harrison, 2023	Phase 3, RCT	NCT04197479	52 weeks	Resmetirom (80 mg, 100 mg), Placebo	Incidence of TEAEs	Biopsy‐confirmed MASH, MRI‐PDFF ≥ 8%, adults ≥ 18 years
Shankar, 2024	Phase 2, RCT	NCT04019561	19 weeks	Cotadutide (300 μg, 600 μg), Placebo	Incidence of TEAEs and serious TEAEs	Biopsy‐confirmed MASLD/MASH, BMI ≥ 30 kg/m^2^, MRI‐PDFF ≥ 10%, adults ≥ 18 years
Sanyal, 2024_a	Phase 2, RCT	NCT04881760	48 weeks	Retatrutide (1 mg, 4 mg, 8 mg, 12 mg), Placebo	Assess mean relative change from baseline in LF	BMI ≥ 30 and ≤ 50 kg/m^2^, MRI‐PDFF ≥ 10%, aged 18–75 years
Sanyal, 2024_b	Phase 2, RCT	NCT04771273	48 weeks	Survodutide (2.4 mg, 4.8 mg, 6.0 mg), Placebo	Histologic improvement in MASH with no worsening of fibrosis	Biopsy‐confirmed MASH, BMI ≥ 25 kg/m^2^, adults aged 18–80 years

Abbreviations: BMI, Body Mass Index; LF, Liver Fat; MASH, Metabolic Dysfunction‐Associated Steatohepatitis; MASLD, Metabolic Dysfunction‐Associated Steatotic Liver Disease; MRI‐PDFF, Magnetic Resonance Imaging Proton Density Fat Fraction; RCT, Randomised Control Trial; TEAEs, Treatment‐Emergent Adverse Event.

### 2.3 Search Strategy

We systematically searched PubMed, Scopus, the Cochrane Central Register of Controlled Trials and clinical trials.gov to identify relevant studies from database inception to January 28th, 2025. The search terms searching all the mentioned databases had the same string including: ((“glucagon receptor agonist” OR “mazdutide” OR “retatrutide” OR “cotadutide” OR “survodutide”) AND (“non‐alcoholic fatty liver disease” OR “NAFLD” OR “NASH” OR “liver” OR “steatohepatitis” OR “metabolic‐associated steatotic liver disease” OR “MASLD”)) OR ((“thyroid hormone receptor beta agonist” OR “resmetirom” OR “MGL‐3196”) AND (“randomized controlled trial” OR “RCT” OR “clinical trial” OR “trial”) AND (“liver fat” OR “fibrosis” OR “steatosis” OR “inflammation” OR “histology” OR “weight loss” OR “MRI‐PDFF” OR “ALT” OR “AST”)). The detailed search strategy is reported in Supplementary S1. No restrictions were applied based on publication status; however, only studies published in English or with available English translations were included.

### 2.4 R Code and Dataset Public Repository

Data was shared using public repository: https://doi.org/10.5281/zenodo.17874754. It can be cited like this: Ayesh, H. (2025). Comparative‐Analysis‐of‐Glucagon‐Receptor‐Agonists‐vs‐Resmetirom‐in‐MASLD‐and‐MASH (Version 1.0.0) [Software]. Zenodo. https://doi.org/10.5281/zenodo.17874755. It has the cleaned study level data with R code to reproduce the results for transparency.

### 2.5 Screening Process

Two reviewers (CA and AA) independently screened titles and abstracts, followed by full‐text reviews to identify eligible studies. Discrepancies were resolved by consensus or by a third reviewer.

### 2.6 Data Extraction and Risk of Bias

Two independent reviewers extracted data using a standardised and piloted form. Collected variables included study baseline characteristics (e.g., age, sex, body weight (kg), body mass index (BMI) (kg/m^2^), AST/ALT levels (IU/L), LDL/HDL levels (mg/dl), waist circumference (cm), Diabetes, HbA1c, MRI‐PDFF, ELF, and adiponectin (mg/L)). Patient demographics, intervention details, outcome measures, information on study design, duration, sample size and adverse events were also extracted (See Tables S3 and S4). Combined means and standard deviations were calculated following the Cochrane Handbook for Systematic Reviews of Interventions Guidelines [18]. To ensure consistency across studies, all AST/ALT levels were standardised to international units per litre (IU/L), LDL/HDL to milligrams per deciliter (mg/dL) and adiponectin to milligrams per litre (mg/L). The rest of the data were incorporated as originally reported. The Cochrane Risk of Bias (ROB) tool was used to assess study quality, with each domain scored as 1 (low), 2 (moderate), or 3 (high) risk [19]. We also evaluated overall confidence in network estimates using the CINeMA (Confidence in Network Meta‐Analysis) framework [20] (See Table S5).

### 2.7 Statistical Analysis

We conducted a frequentist random‐effects network meta‐analysis using the netmeta package in R to evaluate several therapies by analysing data from multiple trials, allowing for both direct and indirect comparisons. This method helps to synthesise data to determine the relative effectiveness of each therapy, even though they were not directly compared in any one trial [18]. Risk ratios (RRs) [17] were used for dichotomous outcomes, and mean differences (MDs) were used for continuous outcomes, each with 95% confidence intervals. Placebo was used as the reference comparator. When multiple doses were available for an intervention, the highest approved dose was used for primary analysis; sensitivity analysis included all doses. Treatment rankings were estimated using P‐scores derived from the netrank function [21] (See Table S8).

### 2.8 Assessment of Heterogeneity, Publication Bias, Inconsistency, and Transitivity

Heterogeneity across studies was assessed using the *I* ^2^ statistic, *τ* ^2^, and Cochran's *Q* test. In regard to thresholds, we interpreted *I* ^2^ values as follows: 0%–25% indicated minimal heterogeneity, 25%–50% moderate heterogeneity, and above 50% high heterogeneity [22]. We pre‐specified to examine heterogeneity by key study characteristics, including age, and sex. The meta and netmeta packages in RStudio were used for all analyses [17]. Treatments were ranked using P‐scores, which estimate the probability that an intervention is among the most effective [21] Global inconsistency was evaluated via design‐by‐treatment interaction models using the decomp.design function. We assessed transitivity by comparing baseline covariates (e.g., age and male sex) across treatment comparisons using ANOVA and boxplot visualizations [23]. Sensitivity analyses included exclusion of high‐risk bias studies and leave‐one‐out analyses to evaluate the influence of individual trials on overall estimates (See Table S10). Publication bias was assessed using funnel plots and Egger's test for the primary outcomes (See Figure S6).

## 3 Results

### 3.1 Study Selection and Characteristics

**FIGURE 1:**
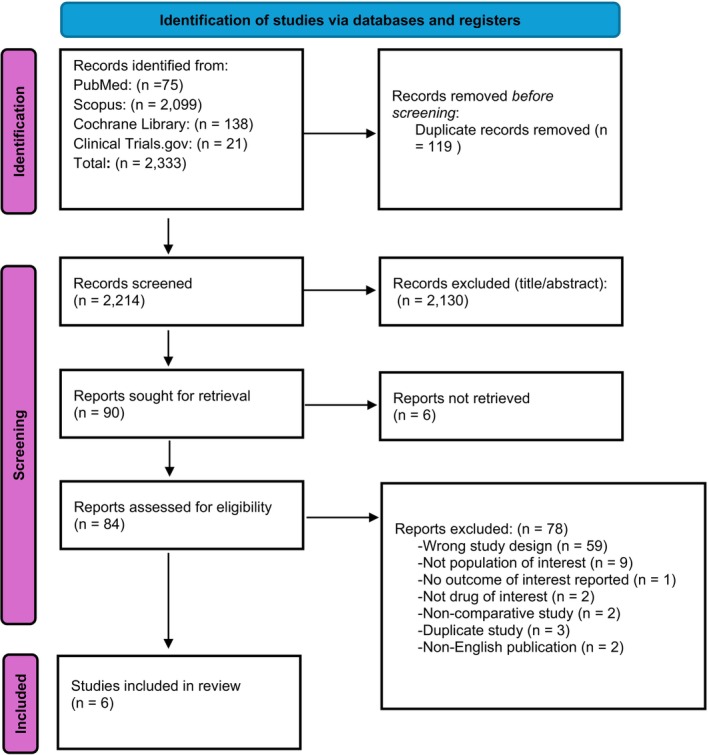
PRISMA flowchart for study selection.

A total of 2333 records were identified through database searching, of which 84 full‐text articles were assessed for eligibility. Six RCTs, encompassing 2528 patients, met inclusion criteria (See Figure 1). These studies compared cotadutide, retarutide, survodutide, resmetirom, and placebo [8, 9, 10, 24, 25, 26]. Study durations ranged from 19 to 52 weeks. Most trials enrolled patients with biopsy‐confirmed MASLD or imaging‐detected hepatic steatosis. The mean age of participants was 52.73 years (SD 11.61), with a mean BMI of 36.27 kg/m^2^ (SD 6.07) and mean waist circumference of 114.05 cm (SD 15.26). Liver function tests showed a mean ALT level of 46.98 IU/L (SD 27.73) and a mean AST level of 34.96 IU/L (SD 18.94). Lipids showed a mean HDL of 32.33 mg/dL (SD 9.33) and mean LDL 78.08 mg/dL (SD 27.99). Glycemic control, measured by HbA1c, had a mean of 6.4% (SD 0.95), with 47.3% of participants being male. ELF and adiponectin had mean and SD of 9.32 (0.69) and 4.40 (2.30) mg/L, subsequently. Baseline characteristics of the included studies and the participants are summarised in Table 1 and Tables S3 and S4.

### 3.2 Biochemical Markers

#### 3.2.1 Changes in ALT

**FIGURE 2:**
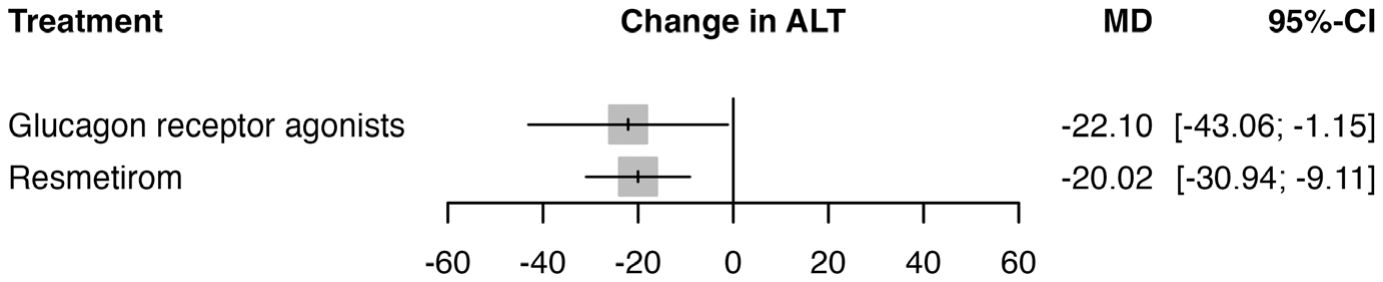
This figure presents a forest plot comparing the efficacy of Glucagon receptor agonists and resmetirom against placebo in patients with MASLD/MASH. The outcome measured is ALT. The mean difference (MD) with 95% confidence interval (CI) shows the likelihood of ALT levels reduction for each treatment compared to placebo. Grey squares represent effect estimates; horizontal lines show 95% CI.

In the random effects model assessing the change in ALT (IU/L), GRAs demonstrated a significant effect with a mean difference (MD) of −22.10 (95% CI: −43.06 to −1.15, *p* = 0.0387) compared to placebo (See Figure 2). Resmetirom also showed a significant reduction in ALT with a MD of −20.02 (95% CI: −30.94 to −9.11, *p* = 0.0003). The heterogeneity analysis revealed high heterogeneity with an *I* ^2^ of 62.5% and *τ* ^2^ = 66.70. Tests of heterogeneity within designs were significant (*Q* = 10.66, df = 4, *p* = 0.0306), indicating variability among the study results. The P‐scores, which rank treatments based on their effectiveness, were highest for GRAs (0.77), followed by resmetirom (0.72) (See Table S8.1). The transitivity assumption was held for age and male gender, with no substantial baseline imbalances in covariates across treatment comparisons. Thus, no major inconsistency was detected. The baseline covariates age and male sex were examined using univariable meta‐regression, but neither was found to significantly contribute to the observed heterogeneity, as indicated by non‐significant moderator *p*‐values. This suggests that residual heterogeneity remains unexplained by these covariates. Sensitivity analyses were conducted to further explore potential sources of heterogeneity. Excluding studies with small sample sizes did not reduce heterogeneity, indicating that sample size was not a contributing factor. Moreover, comparison between fixed and random effects models yielded consistently high heterogeneity, although the overall ranking favouring GRAs remained unchanged. However, leave‐one‐out analysis revealed that the ranking varied with the exclusion of individual studies, suggesting that single studies could influence overall outcomes. This variability indicates that the apparent superiority of GRAs over resmetirom may not be robust and could shift depending on which studies are included. The certainty of evidence was generally very low—low due to concerns of heterogeneity and imprecision (See Table S9.1) and the sensitivity analysis showed stable effect (See Table S10.1). League table summarising pairwise comparisons and rankings is provided in Table S7.1, and the corresponding network plot is shown in Figure S2.1.

#### 3.2.2 Changes in AST

**FIGURE 3:**
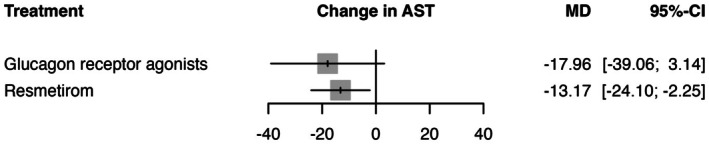
This figure presents a forest plot comparing the efficacy of glucagon receptor agonists and resmetirom against placebo in patients with MASLD/MASH. The outcome measured is AST. The mean difference (MD) with 95% confidence interval (CI) shows the likelihood of AST levels reduction for each treatment compared to placebo. Grey squares represent effect estimates; horizontal lines show 95% CI.

In the random effects model assessing the change in AST (IU/L), resmetirom demonstrated the most significant reduction with a mean difference (MD) of −13.17 (95% CI: −24.10 to −2.25, *p* = 0.0181) compared to placebo (See Figure 3). GRAs showed a nonsignificant reduction with a MD of −17.96 (95% CI: −39.06 to 3.14, *p* = 0.0952). The heterogeneity analysis revealed substantial variability across studies, with an *I* ^2^ of 80.4% and *τ* ^2^ = 82.26. Tests of heterogeneity within designs were significant (*Q* = 20.41, df = 4, *p* < 0.0004), indicating inconsistency among the study results. The P‐scores, which rank treatments based on their effectiveness, were highest for GRAs (0.80), followed by resmetirom (0.67) (See Table S8.2). The transitivity assumption was held for age and male gender, with no substantial baseline imbalances in covariates across treatment comparisons. Thus, no major inconsistency was detected. The baseline covariates age and male sex were examined using univariable meta‐regression, but neither was found to significantly contribute to the observed heterogeneity, as indicated by non‐significant moderator *p*‐values. This suggests that residual heterogeneity remains unexplained by these covariates. Sensitivity analyses were conducted to further explore potential sources of heterogeneity. Excluding studies with small sample sizes did not reduce heterogeneity, indicating that sample size was not a contributing factor. Moreover, comparison between fixed and random effects models yielded consistently high heterogeneity, although the overall ranking favouring GRAs remained unchanged. However, leave‐one‐out analysis revealed that the ranking remained the same with GRAs being superior. We noted in the Harrison, 2024 study that the *I* ^2^ value dropped from approximately 85% in the other results to 0% [10], which supports the robustness of our data, as this variability may indicate random error rather than systematic differences across the studies. The certainty of evidence was generally very low due to concerns of heterogeneity and imprecision (See Table S9.2) and the sensitivity analysis showed stable effect (See Table S10.2). League table summarising pairwise comparisons and rankings is provided in Table S7.2, and the corresponding network plot is shown in Figure S2.2.

#### 3.2.3 Changes in LDL

In the random effects model assessing the change in LDL (mg/dL), resmetirom demonstrated the most significant reduction with a mean difference (MD) of −17.33 (95% CI: −20.54 to −14.13, *p* < 0.0001) compared to placebo. GRAs showed a non‐significant reduction with an MD of −0.3117 (95% CI: −4.20 to 3.58, *p* = 0.8752). The heterogeneity analysis revealed no heterogeneity across studies, with an *I* ^2^ of 0% and *τ* ^2^ = 0. Tests of heterogeneity within designs were non‐significant (*Q* = 2.73, df = 3, *p* = 0.4348), indicating consistency among the study results. The P‐scores, which rank treatments based on their effectiveness, were highest for resmetirom (1.00) followed by GRAs (0.28) and then placebo (0.22) (See Table S8.3). The transitivity assumption was held for age and male gender, with no substantial baseline imbalances in covariates across treatment comparisons. Thus, no major inconsistency was detected. The certainty of evidence was generally low due to concerns of imprecision (See Table S9.3) and the sensitivity analysis showed stable effect (See Table S10.3). League table summarising pairwise comparisons and rankings is provided in Table S7.3, and the corresponding network plot is shown in Figure S2.3.

#### 3.2.4 Changes in HDL

In the random effects model assessing the change in HDL (mg/dl), resmetirom demonstrated a borderline significant increase in HDL with a mean difference (MD) of 2.11 (95% CI: 0.04 to 4.19, *p* = 0.0459) compared to placebo, while GRAs did not show a significant effect with a MD of −0.1871 (95% CI: −1.09 to 0.72, *p* = 0.6851). The heterogeneity analysis revealed no heterogeneity with an *I* ^2^ of 0% and *τ* ^2^ = 0. Tests of heterogeneity within designs were not significant (*Q* = 1.87, df = 3, *p* = 0.5992), indicating no variability among the study results. The P‐scores, which rank treatments based on their effectiveness, were highest for resmetirom (0.98), followed by placebo (0.34) and then GRAs (0.18) (See Table S8.4). The transitivity assumption was held for age and male gender, with no substantial baseline imbalances in covariates across treatment comparisons. Thus, no major inconsistency was detected. The certainty of evidence was generally low due to concerns of imprecision (See Table S9.4), and the sensitivity analysis showed stable effect (See Table S10.4). League table summarising pairwise comparisons and rankings is provided in Table S7.4, and the corresponding network plot is shown in Figure S2.4.

### 3.3 Imaging Outcomes

#### 3.3.1 Change in MRI‐PDFF

**FIGURE 4:**
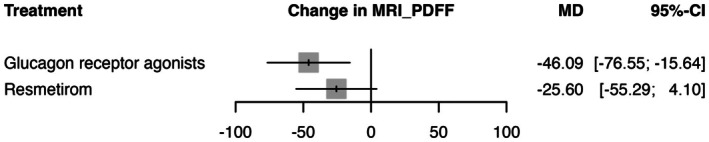
This figure presents a forest plot comparing the efficacy of glucagon receptor agonists and resmetirom against placebo in patients with MASLD/MASH. The outcome measured is Hepatic Fat Fraction (MRI‐PDFF). The mean difference (MD) with 95% confidence interval (CI) shows the likelihood of MRI‐PDFF improvement for each treatment compared to placebo. Grey squares represent effect estimates; horizontal lines show 95% CI.

In the random effects model assessing the change in MRI‐PDFF, GRAs demonstrated the most significant effect with a mean difference (MD) of −46.0924 (95% CI: −76.55 to −15.64, *p* = 0.0030) compared to placebo, indicating a substantial reduction in liver fat content (See Figure 4). Resmetirom showed a non‐significant effect with a MD of −25.5952 (95% CI: −55.29 to 4.10, *p* = 0.0912). The heterogeneity analysis revealed significant variability across studies, with an *I* ^2^ of 98.6% and *τ* ^2^ = 681.23. Tests of heterogeneity within designs were significant (*Q* = 287.08, df = 4, *p* < 0.0001), indicating considerable inconsistency among the study results. The P‐scores, which rank treatments based on their effectiveness, were highest for GRAs (0.91), followed by resmetirom (0.56) and lastly placebo (0.02) (See Table S8.5). The transitivity assumption was held for age and male gender, with no substantial baseline imbalances in covariates across treatment comparisons. Thus, no major inconsistency was detected. To explore potential sources of this heterogeneity, we conducted a univariable meta‐regression analysis and found that age was a confounding variable with a significant *p* = 0.025. The certainty of evidence was generally very low—low due to concerns of heterogeneity and imprecision (See Table S9.5) and the sensitivity analysis showed stable effect (See Table S10.5). League table summarising pairwise comparisons and rankings is provided in Table S7.5, and the corresponding network plot is shown in Figure S2.5.

### 3.4 Histological Outcomes and Surrogate Markers of Fibrosis

#### 3.4.1 Enhanced Liver Fibrosis (ELF)

In the random effects model assessing enhanced liver fibrosis (ELF), GRAs showed a significant decrease in enhanced liver fibrosis with a mean difference (MD) of −1.73 (95% CI: −2.75 to −0.71, *p* = 0.0009) compared to placebo. Resmetirom showed a non‐significant decrease in enhanced liver fibrosis, presenting a MD of −0.26 (95% CI: −0.96 to 0.44, *p* = 0.4675). The heterogeneity analysis revealed high heterogeneity, with *I* ^2^ = 93.4% and *τ* ^2^ = 0.3599. Tests for heterogeneity within designs were significant (*Q* = 45.54, df = 3, *p* = 0.0000), indicating variability among the study results. The P‐scores, which rank treatments based on their likelihood of enhancing liver fibrosis, were highest for GRAs (0.99), followed by resmetirom (0.39) (See Table S8.10). This ranking indicates that GRAs was the highest to decrease enhanced liver fibrosis, followed by resmetirom. The transitivity assumption was held for age and male gender, with no substantial baseline imbalances in covariates across treatment comparisons. Thus, no major inconsistency was detected. The baseline covariates age and male sex were examined using univariable meta‐regression, but both were found to be significantly contributing to the observed heterogeneity, as indicated by *p*‐values of 0.016 for male sex and 0.008 for age. The certainty of evidence was generally very low—low due to concerns of heterogeneity and imprecision (See Table S9.10) and the sensitivity analysis showed stable effect (See Table S10.10). League table summarising pairwise comparisons and rankings is provided in Table S7.10, and the corresponding network plot is shown in Figure S2.10.

#### 3.4.2 Adiponectin

In the random effects model assessing adiponectin (mg/L), GRAs showed a significant increase in adiponectin with a mean difference (MD) of 32.73 (95% CI: 19.27 to 46.20, *p* = 0.0000) compared to placebo. Resmetirom also showed a significant increase in adiponectin, presenting a MD of 0.95 (95% CI: 0.32 to 1.57, *p* = 0.0030). The heterogeneity analysis revealed high heterogeneity, with *I* ^2^ = 73.9% and *τ* ^2^ = 0.2459. Tests for heterogeneity within designs were significant (*Q* = 11.49, df = 3, *p* = 0.0094), indicating variability among the study results. The P‐scores, which rank treatments based on their likelihood of increasing adiponectin, were highest for GRAs (1.00), followed by resmetirom (0.50) (See Table S8.11). This ranking indicates that GRAs had the highest risk of increasing adiponectin, followed by resmetirom. The transitivity assumption was held for age and male gender, with no substantial baseline imbalances in covariates across treatment comparisons. Thus, no major inconsistency was detected. The baseline covariates age and male sex were examined using univariable meta‐regression, but neither was found to significantly contribute to the observed heterogeneity, as indicated by non‐significant moderator *p*‐values. This suggests that residual heterogeneity remains unexplained by these covariates. Sensitivity analyses were conducted to further explore potential sources of heterogeneity. Excluding studies with small sample sizes did not reduce heterogeneity, indicating that sample size was not a contributing factor. Moreover, comparison between fixed and random effects models yielded consistently high heterogeneity. The certainty of evidence was generally low due to concerns of heterogeneity (See Table S9.11) and the sensitivity analysis showed stable effect (See Table S10.11). League table summarising pairwise comparisons and rankings is provided in Table S7.11, and the corresponding network plot is shown in Figure S2.11.

#### 3.4.3 MASH Resolution With No Worsening of Fibrosis

In the random effects model assessing MASH resolution with no worsening of fibrosis, GRAs showed a non‐significant resolution of MASH with a Relative Risk (RR) of 2.42 (95% CI: 0.90 to 6.52, *p* = 0.0799) compared to placebo. Resmetirom showed a significant MASH resolution in risk, presenting a RR of 2.04 (95% CI: 1.02 to 4.11, *p* = 0.0449). The heterogeneity analysis revealed high heterogeneity, with *I* ^2^ = 63.2% and *τ* ^2^ = 0.1735. Tests for heterogeneity within designs were not significant (*Q* = 2.72, df = 1, *p* = 0.0992), indicating consistency among the study results. The P‐scores, which rank treatments based on their likelihood of MASH resolution with no worsening of fibrosis, were highest for GRAs (0.78), followed by resmetirom (0.68) (See Table S8.12). This ranking indicates that GRAs had the highest MASH resolution, followed by resmetirom. The transitivity assumption was held for age and male gender, with no substantial baseline imbalances in covariates across treatment comparisons. Thus, no major inconsistency was detected. The baseline covariates age and male sex were examined using univariable meta‐regression, but neither was found to significantly contribute to the observed heterogeneity, as indicated by non‐significant moderator *p*‐values. Multivariable Meta‐Regression could not be measured because more studies on GRAs are needed. Sensitivity analyses were conducted to further explore potential sources of heterogeneity. Excluding studies with small sample sizes did not reduce heterogeneity, indicating that sample size was not a contributing factor. Moreover, comparison between fixed and random effects models yielded consistently high heterogeneity. The observed discrepancies arise from differences in ranking metrics and the way uncertainty is incorporated in network meta‐analysis. The certainty of evidence was generally very low—low due to concerns of heterogeneity and imprecision (See Table S9.12) and the sensitivity analysis showed stable effect (See Table S10.12). League table summarising pairwise comparisons and rankings is provided in Table S7.12, and the corresponding network plot is shown in Figure S2.12.

### 3.5 Safety Outcomes

#### 3.5.1 Diarrhoea

In the random effects model assessing the incidence of diarrhoea, resmetirom demonstrated a significant increase in the risk with a relative risk (RR) of 2.25 (95% CI: 1.82 to 2.79, *p* < 0.0001) compared to placebo. GRAs also showed a significant increase in the risk of diarrhoea with an RR of 2.30 (95% CI: 1.46 to 3.61, *p* = 0.0003). The heterogeneity analysis revealed no significant heterogeneity, with *I* ^2^ = 0% and *τ* ^2^ = 0. Tests for heterogeneity within designs were not significant (*Q* = 1.81, df = 4, *p* = 0.7702), indicating consistency among the study results. The P‐scores, which rank treatments based on their likelihood of causing diarrhoea, were highest for GRAs (0.77), followed by resmetirom (0.73) (See Table S8.6). The transitivity assumption was held for age and male gender, with no substantial baseline imbalances in covariates across treatment comparisons. Thus, no major inconsistency was detected. The certainty of evidence was generally low—moderate due to concerns of imprecision (See Table S9.6), and the sensitivity analysis showed stable effect (See Table S10.6). League table summarising pairwise comparisons and rankings is provided in Table S7.6, and the corresponding network plot is shown in Figure S2.6.

#### 3.5.2 Fatigue

In the random effects model assessing the incidence of fatigue, resmetirom demonstrated no significant effect in the risk of fatigue with a relative risk (RR) of 0.93 (95% CI: 0.62 to 1.38, *p* = 0.7107) compared to placebo. GRAs also did not show a significant effect in the risk of fatigue with an RR of 2.11 (95% CI: 0.89 to 4.98, *p* = 0.0884). The heterogeneity analysis revealed no significant heterogeneity, with *I* ^2^ = 0% and *τ* ^2^ = 0. Tests for heterogeneity within designs were not significant (*Q* = 1.69, df = 3, *p* = 0.6399), indicating consistency among the study results. The P‐scores, which rank treatments based on their likelihood of causing fatigue, were highest for resmetirom (0.80), followed by placebo (0.66) and then GRAs (0.04) (See Table S8.7). The transitivity assumption held for age and male gender, with no substantial baseline imbalances in covariates across treatment comparisons. Thus, no major inconsistency was detected. The certainty of evidence was generally moderate (See Table S9.8) and the sensitivity analysis showed stable effect (See Table S10.8). League table summarising pairwise comparisons and rankings is provided in Table S7.8, and the corresponding network plot is shown in Figure S2.8.

#### 3.5.3 Nausea

In the random effects model assessing the incidence of nausea, GRAs showed the highest significant increase in risk with a relative risk (RR) of 3.53 (95% CI: 2.17 to 5.74, *p* < 0.0001) compared to placebo. Resmetirom followed with a significant increase in risk, presenting an RR of 1.90 (95% CI: 1.33 to 2.70, *p* = 0.0004). The heterogeneity analysis indicated mild heterogeneity with *I* ^2^ = 20.1% and *τ* ^2^ = 0.03. Tests for heterogeneity within designs were not significant (*Q* = 5.01, df = 4, *p* = 0.2865), suggesting consistency among the study results. The P‐scores, which rank treatments based on their likelihood of causing nausea, were highest for placebo (1.00), followed by resmetirom (0.49) and GRAs (0.01) (See Table S8.8). The transitivity assumption held for age and male gender, with no substantial baseline imbalances in covariates across treatment comparisons. Thus, no major inconsistency was detected. The certainty of evidence was generally moderate (See Table S9.7) and the sensitivity analysis showed stable effect (See Table S10.7). League table summarising pairwise comparisons and rankings is provided in Table S7.7, and the corresponding network plot is shown in Figure S2.7.

#### 3.5.4 Serious Adverse Events

In the random effects model assessing serious adverse events, GRAs showed no significant increase in risk with a relative risk (RR) of 1.15 (95% CI: 0.43 to 3.06, *p* = 0.7854) compared to placebo. Resmetirom also showed no significant increase in risk, presenting a MD of 1.13 (95% CI: 0.81 to 1.57, *p* = 0.4644). The heterogeneity analysis revealed no significant heterogeneity, with *I* ^2^ = 0% and *τ* ^2^ = 0. Tests for heterogeneity within designs were not significant (*Q* = 0.07, df = 4, *p* = 0.9994), indicating consistency among the study results. The P‐scores, which rank treatments based on their likelihood of causing serious adverse events, were highest for resmetirom (0.63), followed by GRAs (0.56) and then placebo (0.31) (See Table S8.9). This ranking indicates that resmetirom had the highest risk of serious adverse events, followed by GRAs compared to placebo. The transitivity assumption was held for age and male gender, with no substantial baseline imbalances in covariates across treatment comparisons. Thus, no major inconsistency was detected. The certainty of evidence was generally low–moderate due to concerns of imprecision (See Table S9.9), and the sensitivity analysis showed stable effect (See Table S10.9). League table summarising pairwise comparisons and rankings is provided in Table S7.9, and the corresponding network plot is shown in Figure S2.9.

## 4 Discussion

This network meta‐analysis demonstrated significant clinical insights into the efficacy of GRAs and resmetirom for treating patients with MASLD/MASH compared to placebo. GRAs showed the highest effectiveness in terms of ALT reduction and significant reduction for MRI‐PDFF, highlighting its potential in managing liver fat reduction. But it also showed a significant decrease in enhanced liver fibrosis. On the other hand, resmetirom demonstrated substantial efficacy, particularly in improving AST levels, reducing HDL and LDL levels, and improving MASH resolution without worsening of fibrosis. However, both GRAs and resmetirom showed significant reductions in ALT levels and significant increase in adiponectin. Adverse events analysis indicated that GRAs were associated with higher risks of nausea and diarrhoea, with resmetirom offering a more favourable safety profile. GRAs ranked first among ALT, AST, MRI‐PDFF, diarrhoea, ELF, adiponectin, and MASH resolution with no worsening of fibrosis, whereas resmetirom ranked first for HDL, LDL, fatigue, and serious adverse events. The overall quality of evidence for this comparison was high, supported by consistent findings across multiple studies with low risk of bias. These findings support the clinical utility of GRAs as a promising pharmacologic option for MASLD/MASH.

To our knowledge, this study is the first to highlight the significance of efficacy and safety and to provide a practical comparison of GRAs and resmetirom for clinical practice. Our analysis provides a detailed assessment of different outcomes, including changes in biochemical markers such as ALT, AST, HDL, LDL, and liver fat reduction. It also provides information about some of the common and serious adverse events, all of which need to be taken into consideration when selecting a therapeutic regimen. Additionally, it highlights some histological outcomes including ELF, adiponectin, and MASH resolution with no worsening of fibrosis.

The observed benefits of resmetirom on liver enzymes and hepatic fat improvement are clinically meaningful, especially considering the limited treatment options currently available for MASLD/MASH. These benefits are largely attributed to the mechanism of action of resmetirom as a selective thyroid hormone receptor‐β agonist, which promotes improvements in liver enzymes (ALT, AST) and reduces liver fat [9]. Although not particularly sensitive [27], markers of hepatic cytolysis, specifically, ALT and AST serve as secondary indicators of possible liver inflammation in patients with NAFLD [28]. Thus, GRAs, through their primary mechanism of action, not only stimulate insulin release from beta cells in the presence of high blood glucose and reduce glucagon secretion from alpha cells, but also improve ALT levels [29]. These multidimensional benefits are particularly relevant in MASLD/MASH, which frequently coexists with obesity and type 2 diabetes. Our findings may inform the prioritisation of GRAs as a preferred agent in MASLD management, particularly in patients with high ALT levels.

Despite the favourable safety profile and beneficial effects of resmetirom, concerns remain regarding its appropriate prescription. The targeted population for resmetirom includes patients with histologically confirmed MASH and fibrosis stages 2 and 3, excluding those with early‐stage fibrosis (stages 0 and 1) and cirrhosis. A diagnosis of MASLD must first be established before selecting eligible patients for treatment. To address this, noninvasive diagnostic strategies have been defined [30]. However, there are concerns regarding these noninvasive tests, as they do not always accurately identify the appropriate patient population. Thus, liver biopsy remains a potentially important diagnostic option. In fact, due to the difficulty of NITs in determining disease stage, both overuse and underuse remain common clinical challenges. For example, differentiating between stage F3 fibrosis and early stage F4 (cirrhosis) using NITs remains difficult, and misclassification between these stages is therefore relatively common. The thresholds defined for the NITs also have a significant impact on the selected patient population [2]. A study reported by Lazarus et al. showed that 40% of patients who were eligible to be prescribed resmetirom did not meet the histological criteria, while more than 50% of histologically eligible patients were under‐prescribed. Moreover, the cost‐effectiveness of the drug is another concern [31]. Resmetirom costs approximately $1500 per month, representing a significant burden on healthcare budgets, with the estimated lifetime treatment cost reaching $348,432 compared to $281,668 for placebo. However, cost‐effectiveness analyses suggest that resmetirom may reduce complications associated with advanced liver disease and improve quality‐adjusted life years (QALYs). Therefore, incorporating resmetirom into local healthcare plans could prove to be both cost‐effective and clinically beneficial [2].

GRAs have shown greater efficacy in improving glycemic control and reducing body weight compared to GLP‐1RAs alone. Glucagon receptor activation increases energy expenditure, which leads to weight loss and also improves lipid metabolism by lowering triglyceride levels in the blood and liver, as well as reducing plasma cholesterol. Glucagon receptor expression is highest in hepatocytes and limited in adipose tissue. Therefore, its metabolic effects are primarily hepatic, which includes fatty acid oxidation, gluconeogenesis, and glycogenolysis. But at the same time, it also promotes lipolysis indirectly by increasing fat mobilisation from peripheral stores. GLP‐1 receptor activation complements these effects by reducing caloric intake. Thus, dual receptor activation may amplify therapeutic benefits in conditions like MASH, liver fibrosis, and other features of metabolic syndrome. The improvements in liver fat and inflammation appear to result from both direct hepatic effects and secondary benefits of weight loss [32].

At this time, GRAs are not approved for the treatment of MASLD/MASH. However, they are being actively studied in ongoing clinical trials, with early findings showing promising results. Our findings are consistent with previous pairwise meta‐analyses that have reported beneficial effects of GRAs on ALT levels in MASLD. However, prior studies have been limited by the lack of head‐to‐head comparisons among active pharmacologic agents. By incorporating both direct and indirect evidence, this network meta‐analysis provides a more comprehensive understanding of the relative efficacy of these treatments. Notably, the superior ranking of GRAs aligns with recent RCTs such as the ESSENCE trial [33], though our synthesis offers enhanced statistical power and broader comparisons. In contrast to older meta‐analyses, our results also suggest that newer GRA agents may be more effective than the older ones.

This study has several notable strengths. We conducted a comprehensive literature search across major databases and included only RCTs, enhancing the internal validity of our findings. The use of a frequentist network meta‐analysis allowed us to simultaneously compare multiple interventions and rank them based on efficacy. We applied robust methodological tools, including the CINeMA framework to evaluate the certainty of evidence and a structured transitivity assessment using baseline covariate balance [20]. Our sensitivity analyses, including exclusion of high‐risk studies and leave‐one‐out tests, confirmed the stability of our conclusions.

Despite these strengths, our analysis has limitations. The outcomes assessed are surrogate markers rather than direct clinical endpoints. Another limitation is that heterogeneity was moderate in several comparisons, reflecting differences in trial populations, intervention dosing, and outcome definitions. Although most included studies had a low‐some concerns risk of bias, a few trials lacked detailed reporting on allocation concealment and adherence. Additionally, the network structure was anchored by placebo in all comparisons. Publication bias could not be completely ruled out due to limited funnel plot asymmetry tests for small networks. While our network meta‐analysis identifies promising pharmacologic strategies for MASLD, several gaps remain. Future research should prioritise head‐to‐head RCTs comparing top‐ranked agents such as cotadutide, retarutide, and survodutide to better define their relative efficacy and tolerability. Studies with longer follow‐up periods are also needed to assess sustained histological improvement and long‐term liver outcomes. Furthermore, trials focused on specific patient subgroups—such as those with diabetes, advanced fibrosis, or lean MASLD—would enhance the generalisability and clinical applicability of future evidence. Finally, more consistent reporting of metabolic and histological endpoints would improve comparability across studies and strengthen future meta‐analytic efforts.

## 5 Conclusion

This network meta‐analysis found that GRAs superiorly reduce ALT levels, MRI‐PDFF, and ELF. Resmetirom significantly reduces AST along with HDL and LDL levels, increases MASH resolution without worsening of fibrosis, and it offers a more favourable safety profile. Both GRAs and resmetirom significantly increase adiponectin. These findings emphasise the promise of both therapeutic classes and highlight the need for further comparative trials to inform treatment decisions.

## Author Contributions

**Celina R. Andonie:** conceptualization, methodology, writing – original draft, writing – review and editing, formal analysis, project administration, investigation. **Alaaeddin Abusalameh:** conceptualization, methodology, investigation, writing – original draft, writing – review and editing, project administration. **Ibrahim Ismail:** methodology, conceptualization, project administration, and review of final draft. **Tamer Hodrob:** methodology, project administration, and review of final draft. **Mahmoud Ladadweh:** methodology, review of final draft, conceptualization, and project administration. **Hazem Ayesh:** methodology, writing – review and editing, formal analysis, and project administration. All authors have read and agreed to the published version of the manuscript.

## Funding

The authors has nothing to report.

## Ethics Statement

The authors have nothing to report.

## Consent

The authors have nothing to report.

## Conflicts of Interest

The authors declare no conflicts of interest.

## Supporting information


**Supplementary S1:** Detailed search strategy.
**Figure S2:** Network plots of treatment comparisons.
**Figure S2:1:** Network plot of treatment comparisons for changes in ALT.
**Figure S2:2:** Network plot of treatment comparisons for changes in AST.
**Figure S2:3:** Network plot of treatment comparisons for changes in LDL.
**Figure S2:4:** Network plot of treatment comparisons for changes in HDL.
**Figure S2:5:** Network plot of treatment comparisons for hepatic fat reduction (MRI‐PDFF).
**Figure S2:6:** Network plot of treatment comparisons for diarrhoea.
**Figure S2:7:** Network plot of treatment comparisons for fatigue.
**Figure S2:8:** Network plot of treatment comparisons for nausea.
**Figure S2:9:** Network plot of treatment comparisons for serious adverse events.
**Figure S2:10:** Network plot of treatment comparisons for enhanced liver fibrosis.
**Figure S2:11:** Network plot of treatment comparisons for adiponectin.
**Figure S2:12:** Network plot of treatment comparisons for MASH resolution with no worsening of fibrosis.
**Table S3:** Baseline characteristic for the included studies.
**Table S4:** Baseline characteristics of the participants.
**Table S5:** Risk of bias table.
**Figure S6:** Publication bias (funnel plot).
**Table S7:1:** ALT league.
**Table S7:2:** AST league.
**Table S7:3:** LDL league.
**Table S7:4:** HDL league.
**Table S7:5:** Hepatic fat reduction (MRI‐PDFF) league.
**Table S7:6:** Diarrhoea league.
**Table S7:7:** Fatigue league.
**Table S7:8:** Nausea league.
**Table S7:9:** Serious adverse events league.
**Table S7:10:** Enhanced liver fibrosis (ELF) league.
**Table S7:11:** Adiponectin league.
**Table S7:12:** MASH resolution with no worsening of fibrosis league.
**Table S8:** Treatment ranking for each outcome.
**Table S8:1:** Treatment ranking for the change in ALT.
**Table S8:2:** Treatment ranking for the change in AST.
**Table S8:3:** Treatment ranking for the change in LDL.
**Table S8:4:** Treatment ranking for the change in HDL.
**Table S8:5:** Treatment ranking for the change in hepatic fat reduction (MRI‐PDFF).
**Table S8:6:** Treatment ranking for diarrhoea.
**Table S8:7:** Treatment ranking for fatigue.
**Table S8:8:** Treatment ranking for nausea.
**Table S8:9:** Treatment ranking for serious adverse events.
**Table S8:10:** Treatment ranking for enhanced liver fibrosis (ELF).
**Table S8:11:** Treatment ranking for adiponectin.
**Table S8:12:** Treatment ranking for MASH resolution with no worsening of fibrosis.
**Table S9:1:** Certainty of evidence for change in ALT.
**Table S9:2:** Certainty of evidence for change in AST.
**Table S9:3:** Certainty of evidence for change in LDL.
**Table S9:4:** Certainty of evidence for change in HDL.
**Table S9:5:** Certainty of evidence for change in hepatic fat reduction (MRI‐PDFF).
**Table S9:6:** Certainty of evidence for diarrhoea.
**Table S9:7:** Certainty of evidence for fatigue.
**Table S9:8:** Certainty of evidence for nausea.
**Table S9:9:** Certainty of evidence for serious adverse events.
**Table S9:10:** Certainty of evidence for enhanced liver fibrosis (ELF).
**Table S9:11:** Certainty of evidence for adiponectin.
**Table S9:12:** Certainty of evidence for MASH resolution with no worsening of fibrosis.
**Table S10:** Sensitivity analysis for each outcome.
**Table S10:1:** Leave‐one‐out‐analysis for ALT.
**Table S10:2:** Leave‐one‐out‐analysis for AST.
**Table S10:3:** Leave‐one‐out‐analysis for LDL.
**Table S10:4:** Leave‐one‐out‐analysis for HDL.
**Table S10:5:** Leave‐one‐out‐analysis for hepatic fat reduction (MRI‐PDFF).
**Table S10:6:** Leave‐one‐out‐analysis for diarrhoea.
**Table S10:7:** Leave‐one‐out‐analysis for fatigue.
**Table S10:8:** Leave‐one‐out‐analysis for nausea.
**Table S10:9:** Leave‐one‐out‐analysis for serious adverse events.
**Table S10:10:** Leave‐one‐out‐analysis for enhanced liver fibrosis (ELF).
**Table S10:11:** Leave‐one‐out‐analysis for adiponectin.
**Table S10:12:** Leave‐one‐out‐analysis for MASH resolution with no worsening of fibrosis.
**Supplementary S11:** PRISMA checklist.

## Data Availability

The data that support the findings of this study are openly available in Zenodo at https://doi.org/10.5281/zenodo.17874754.

## References

[1] M. E. Rinella , J. V. Lazarus , V. Ratziu , et al., “A Multisociety Delphi Consensus Statement on New Fatty Liver Disease Nomenclature,” Hepatology 78, no. 6 (2023): 1966–1986, 10.1097/HEP.0000000000000520. 37363821 PMC10653297

[2] E. Kaya , S. Aksoy , N. Oruc , et al., “Non‐Invasive Tests for Resmetirom Treatment Fail to Accurately Define the Target Population: Evidence From a Biopsy‐Proven MASLD Cohort,” Hepatology Forum 6, no. 3 (2025): 111–115, 10.14744/hf.2025.2025.0050. 40686597 PMC12268769

[3] Z. M. Younossi , P. Golabi , J. Paik , et al., “Prevalence of Metabolic Dysfunction‐Associated Steatotic Liver Disease in the Middle East and North Africa,” Liver International 44, no. 4 (2024): 1061–1070, 10.1111/liv.15852. 38305642

[4] S. L. Friedman , B. A. Neuschwander‐Tetri , M. Rinella , and A. J. Sanyal , “Mechanisms of NAFLD Development and Therapeutic Strategies,” Nature Medicine 24, no. 7 (2018): 908–922, 10.1038/s41591-018-0104-9. PMC6553468 29967350

[5] H. Ayesh , A. Beran , S. Suhail , S. Ayesh , and K. Niswender , “Comparative Analysis of Resmetirom vs. FGF21 Analogs vs. GLP‐1 Agonists in MASLD and MASH: Network Meta‐Analysis of Clinical Trials,” Biomedicine 12, no. 10 (2024): 2328, 10.3390/biomedicines12102328. PMC11505228 39457640

[6] X. Xu , K. L. Poulsen , L. Wu , et al., “Targeted Therapeutics and Novel Signaling Pathways in Non‐Alcohol‐Associated Fatty Liver/Steatohepatitis (NAFL/NASH),” Signal Transduction and Targeted Therapy 7, no. 1 (2022): 287, 10.1038/s41392-022-01119-3. 35963848 PMC9376100

[7] M. Kokkorakis , C. Boutari , M. A. Hill , et al., “Resmetirom, the First Approved Drug for the Management of Metabolic Dysfunction‐Associated Steatohepatitis: Trials, Opportunities, and Challenges,” Metabolism 154 (2024): 155835, 10.1016/j.metabol.2024.155835. 38508373

[8] S. A. Harrison , R. Taub , G. W. Neff , et al., “Resmetirom for Nonalcoholic Fatty Liver Disease: A Randomized, Double‐Blind, Placebo‐Controlled Phase 3 Trial,” Nature Medicine 29, no. 11 (2023): 2919–2928, 10.1038/s41591-023-02603-1. PMC10667098 37845512

[9] S. A. Harrison , M. R. Bashir , C. D. Guy , et al., “Resmetirom (MGL‐3196) for the Treatment of Non‐Alcoholic Steatohepatitis: A Multicentre, Randomised, Double‐Blind, Placebo‐Controlled, Phase 2 Trial,” Lancet 394, no. 10213 (2019): 2012–2024, 10.1016/S0140-6736(19)32517-6. 31727409

[10] S. A. Harrison , P. Bedossa , C. D. Guy , et al., “A Phase 3, Randomized, Controlled Trial of Resmetirom in NASH With Liver Fibrosis,” New England Journal of Medicine 390, no. 6 (2024): 497–509, 10.1056/nejmoa2309000. 38324483

[11] D. Zhou and J. Fan , “Drug Treatment for Metabolic Dysfunction‐Associated Steatotic Liver Disease: Progress and Direction,” Chinese Medical Journal 137, no. 22 (2024): 2687–2696, 10.1097/CM9.0000000000003355. 39470028 PMC11611247

[12] A. M. Mousa , M. Mahmoud , and G. M. AlShuraiaan , “Resmetirom: The First Disease‐Specific Treatment for MASH,” International Journal of Endocrinology 2025 (2025): 6430023, 10.1155/ije/6430023. 40212963 PMC11985219

[13] P. N. Newsome , K. Buchholtz , K. Cusi , et al., “A Placebo‐Controlled Trial of Subcutaneous Semaglutide in Nonalcoholic Steatohepatitis,” New England Journal of Medicine 384, no. 12 (2021): 1113–1124, 10.1056/NEJMoa2028395. 33185364

[14] M. J. Armstrong , P. Gaunt , G. P. Aithal , et al., “Liraglutide Safety and Efficacy in Patients With Non‐Alcoholic Steatohepatitis (LEAN): A Multicentre, Double‐Blind, Randomised, Placebo‐Controlled Phase 2 Study,” Lancet 387, no. 10019 (2016): 679–690, 10.1016/S0140-6736(15)00803-X. 26608256

[15] M. J. Page , J. E. McKenzie , P. M. Bossuyt , et al., “The PRISMA 2020 Statement: An Updated Guideline for Reporting Systematic Reviews,” BMJ 372 (2021): n71, 10.1136/bmj.n71. 33782057 PMC8005924

[16] C. R. Andonie , “OSF Registries | Comparative Analysis of Glucagon Receptor Agonists vs Resmetirom in MASLD and MASH: Network Meta‐Analysis of Clinical Trials,” (2025), https://osf.io/e4j5f.10.1002/edm2.7015741466530

[17] S. Balduzzi , G. Rücker , A. Nikolakopoulou , et al., “Netmeta: An R Package for Network Meta‐Analysis Using Frequentist Methods,” Journal of Statistical Software 106, no. 2 (2023): 2, 10.18637/jss.v106.i02.

[18] Cochrane Training , “Cochrane Handbook for Systematic Reviews of Interventions,” 10.1097/CM9.0000000000003355.

[19] J. A. C. Sterne , J. Savović , M. J. Page , et al., “RoB 2: A Revised Tool for Assessing Risk of Bias in Randomised Trials,” BMJ 366 (2019): l4898, 10.1136/bmj.l4898. 31462531

[20] A. Nikolakopoulou , J. P. T. Higgins , T. Papakonstantinou , et al., “CINeMA: An Approach for Assessing Confidence in the Results of a Network Meta‐Analysis,” PLoS Medicine 17, no. 4 (2020): e1003082, 10.1371/journal.pmed.1003082. 32243458 PMC7122720

[21] G. Rücker and G. Schwarzer , “Ranking Treatments in Frequentist Network Meta‐Analysis Works Without Resampling Methods,” BMC Medical Research Methodology 15, no. 1 (2015): 58, 10.1186/s12874-015-0060-8. 26227148 PMC4521472

[22] J. P. T. Higgins , “Measuring Inconsistency in Meta‐Analyses,” BMJ 327, no. 7414 (2003): 557–560, 10.1136/bmj.327.7414.557. 12958120 PMC192859

[23] R. L. Nuzzo , “The Box Plots Alternative for Visualizing Quantitative Data,” PM & R: The Journal of Injury, Function, and Rehabilitation 8, no. 3 (2016): 268–272, 10.1016/j.pmrj.2016.02.001. 26892802

[24] S. S. Shankar , S. J. Daniels , D. Robertson , et al., “Safety and Efficacy of Novel Incretin co‐Agonist Cotadutide in Biopsy‐Proven Noncirrhotic MASH With Fibrosis,” Clinical Gastroenterology and Hepatology 22, no. 9 (2024): 1847–1857.e11, 10.1016/j.cgh.2024.04.017. 38729399

[25] A. J. Sanyal , L. M. Kaplan , J. P. Frias , et al., “Triple Hormone Receptor Agonist Retatrutide for Metabolic Dysfunction‐Associated Steatotic Liver Disease: A Randomized Phase 2a Trial,” Nature Medicine 30, no. 7 (2024): 2037–2048, 10.1038/s41591-024-03018-2. PMC11271400 38858523

[26] A. J. Sanyal , P. Bedossa , M. Fraessdorf , et al., “A Phase 2 Randomized Trial of Survodutide in MASH and Fibrosis,” New England Journal of Medicine 391, no. 4 (2024): 311–319, 10.1056/nejmoa2401755. 38847460

[27] P. Portillo‐Sanchez , F. Bril , M. Maximos , et al., “High Prevalence of Nonalcoholic Fatty Liver Disease in Patients With Type 2 Diabetes Mellitus and Normal Plasma Aminotransferase Levels,” Journal of Clinical Endocrinology and Metabolism 100, no. 6 (2015): 2231–2238, 10.1210/jc.2015-1966. 25885947 PMC6287506

[28] M. Ekstedt , L. E. Franzén , U. L. Mathiesen , et al., “Long‐Term Follow‐Up of Patients With NAFLD and Elevated Liver Enzymes,” Hepatology 44, no. 4 (2006): 865–873, 10.1002/hep.21327. 17006923

[29] T. Salvatore , R. Nevola , P. C. Pafundi , et al., “Incretin Hormones: The Link Between Glycemic Index and Cardiometabolic Diseases,” Nutrients 11, no. 8 (2019): 1878, 10.3390/nu11081878. 31412576 PMC6724226

[30] M. Noureddin , M. R. Charlton , S. A. Harrison , et al., “Expert Panel Recommendations: Practical Clinical Applications for Initiating and Monitoring Resmetirom in Patients With MASH/NASH and Moderate to Noncirrhotic Advanced Fibrosis,” Clinical Gastroenterology and Hepatology 22, no. 12 (2024): 2367–2377, 10.1016/j.cgh.2024.07.003. 39038768

[31] J. V. Lazarus , “The Inherent Value of Treating Metabolic Dysfunction–Associated Steatohepatitis,” JAMA Network Open 8, no. 6 (2025): e2517129, 10.1001/jamanetworkopen.2025.17129. 40577021

[32] E. Kaya , Y. Yilmaz , and N. Alkhouri , “Survodutide in MASH: Bridging the Gap Between Hepatic and Systemic Metabolic Dysfunction,” Expert Opinion on Investigational Drugs 33, no. 12 (2024): 1167–1176, 10.1080/13543784.2024.2441865. 39663847

[33] P. N. Newsome , A. J. Sanyal , K. A. Engebretsen , et al., “Semaglutide 2.4 Mg in Participants With Metabolic Dysfunction‐Associated Steatohepatitis: Baseline Characteristics and Design of the Phase 3 ESSENCE Trial,” Alimentary Pharmacology & Therapeutics 60, no. 11–12 (2024): 1525–1533, 10.1111/apt.18331. 39412509 PMC11599791

