# Supplementary material for: Comparative Analysis of Glucagon Receptor Agonists vs. Resmetirom in MASLD and MASH: Network Meta‐Analysis of Clinical Trials
Source: Endocrinol Diabetes Metab. 2025 Dec 30;9(1):e70157. doi: 10.1002/edm2.70157 (PMC12750095; doi:10.1002/edm2.70157)
Supplement: Supplementary file 1 — Supplementary S1: Detailed search strategy. Figure S2: Network plots of treatment comparisons. Figure S2:1: Network plot of treatment comparisons for changes in ALT. Figure S2:2: Network plot of treatment comparisons for changes in AST. Figure S2:3: Network plot of treatment comparisons for changes in LDL. Figure S2:4: Network plot of treatment comparisons for changes in HDL. Figure S2:5: Network plot of treatment comparisons for hepatic fat reduction (MRI‐PDFF). Figure S2:6: Network plot of treatment comparisons for diarrhoea. Figure S2:7: Network plot of treatment comparisons for fatigue. Figure S2:8: Network plot of treatment comparisons for nausea. Figure S2:9: Network plot of treatment comparisons for serious adverse events. Figure S2:10: Network plot of treatment comparisons for enhanced liver fibrosis. Figure S2:11: Network plot of treatment comparisons for adiponectin. Figure S2:12: Network plot of treatment comparisons for MASH resolution with no worsening of fibrosis. Table S3: Baseline characteristic for the included studies. Table S4: Baseline characteristics of the participants. Table S5: Risk of bias table. Figure S6: Publication bias (funnel plot). Table S7:1: ALT league. Table S7:2: AST league. Table S7:3: LDL league. Table S7:4: HDL league. Table S7:5: Hepatic fat reduction (MRI‐PDFF) league. Table S7:6: Diarrhoea league. Table S7:7: Fatigue league. Table S7:8: Nausea league. Table S7:9: Serious adverse events league. Table S7:10: Enhanced liver fibrosis (ELF) league. Table S7:11: Adiponectin league. Table S7:12: MASH resolution with no worsening of fibrosis league. Table S8: Treatment ranking for each outcome. Table S8:1: Treatment ranking for the change in ALT. Table S8:2: Treatment ranking for the change in AST. Table S8:3: Treatment ranking for the change in LDL. Table S8:4: Treatment ranking for the change in HDL. Table S8:5: Treatment ranking for the change in hepatic fat reduction (MRI‐PDFF). Table S8:6: Treatment ranking for diarrhoea. Table [file EDM2-9-e70157-s001.docx]

Table of Contents

[Supplement S1. Search strategy 6](#_Toc213429614)

[Supplement S2. Network plots of treatment comparisons 7](#_Toc213429615)

[Figure S2.1: Network Plot of Treatment Comparisons for Changes in ALT 7](#_Toc213429616)

[Figure S2.2: Network Plot of Treatment Comparisons for Changes in AST 8](#_Toc213429617)

[Figure S2.3: Network Plot of Treatment Comparisons for Changes in LDL 9](#_Toc213429618)

[Figure S2.4: Network Plot of Treatment Comparisons for Changes in HDL 10](#_Toc213429619)

[Figure S2.5: Network Plot of Treatment Comparisons for Hepatic Fat Reduction (MRI-PDFF) 11](#_Toc213429620)

[Figure S2.6: Network Plot of Treatment Comparisons for Diarrhea 12](#_Toc213429621)

[Figure S2.7: Network Plot of Treatment Comparisons for Nausea 13](#_Toc213429622)

[Figure S2.8: Network Plot of Treatment Comparisons for Fatigue 14](#_Toc213429623)

[Figure S2.9: Network Plot of Treatment Comparisons for Serious Adverse Events 15](#_Toc213429624)

[Figure S2.10: Network Plot of Enhanced Liver Fibrosis (ELF) 16](#_Toc213429625)

[Figure S2.11: Network Plot of Adiponectin 17](#_Toc213429626)

[Figure S2.12: Network Plot of MASH Resolution with No Worsening of Fibrosis 18](#_Toc213429627)

[Supplement S3 Baseline characteristics of the included studies 19](#_Toc213429628)

[Supplement S4. Baseline characteristics of the participants 22](#_Toc213429629)

[Abbreviations: 22](#_Toc213429630)

[Supplement S5. Risk of bias table 23](#_Toc213429631)

[Supplement S6. Publication bias (funnel plot) 24](#_Toc213429632)

[Figure S6.1: Change in ALT 24](#_Toc213429633)

[Figure S6.2: Change in AST 25](#_Toc213429634)

[Figure S6.3: Change in LDL 26](#_Toc213429635)

[Figure S6.4: Change in HDL 27](#_Toc213429636)

[Figure S6.5: Hepatic Fact Reduction (MRI-PDFF) 28](#_Toc213429637)

[Figure S6.6: Diarrhea 29](#_Toc213429638)

[Figure S6.7: Nausea 30](#_Toc213429639)

[Figure S6.8: Fatigue 31](#_Toc213429640)

[Figure S6.9: Serious Adverse Events 32](#_Toc213429641)

[Figure S6.10: Enhanced Liver Fibrosis (ELF) 33](#_Toc213429642)

[Figure S6.11: Adiponectin 34](#_Toc213429643)

[Figure S6.12: MASH Resolution with No Worsening in Fibrosis 35](#_Toc213429644)

[Supplement S7: League Tables 36](#_Toc213429645)

[Table S7.1: ALT League 36](#_Toc213429646)

[Table S7.2: AST League 36](#_Toc213429647)

[Table S7.3: LDL League 36](#_Toc213429648)

[Table S7.4: HDL League 36](#_Toc213429649)

[Table S7.5: Hepatic Fat Reduction (MRI-PDFF) League 36](#_Toc213429650)

[Table S7.6: Diarrhea League 37](#_Toc213429651)

[Table S7.7: Nausea League 37](#_Toc213429652)

[Table S7.8: Fatigue League 37](#_Toc213429653)

[Table S7.9: Serious Adverse Events League 37](#_Toc213429654)

[Table S7.10: Enhanced Liver Fibrosis (ELF) League 37](#_Toc213429655)

[Table S7.11: Adiponectin League 38](#_Toc213429656)

[Table S7.12: MASH Resolution with No Worsening in Fibrosis League 38](#_Toc213429657)

[Supplement S8: Treatment ranking for each outcome 39](#_Toc213429658)

[Table S8.1: Change in ALT 39](#_Toc213429659)

[Table S8.2: Change in AST 39](#_Toc213429660)

[Table S8.3: Change in LDL 39](#_Toc213429661)

[Table S8.4: Change in HDL 39](#_Toc213429662)

[Table S8.5: Change in Hepatic Fat Fraction (MRI-PDFF) 39](#_Toc213429663)

[Table S8.6: Diarrhea 40](#_Toc213429664)

[Table S8.7: Nausea 40](#_Toc213429665)

[Table S8.8: Fatigue 40](#_Toc213429666)

[Table S8.9: Serious Adverse Events 40](#_Toc213429667)

[Table S8.10: Enhanced Liver Fibrosis (ELF) 40](#_Toc213429668)

[Table S8.11: Adiponectin 41](#_Toc213429669)

[Table S8.12: MASH Resolution with No Worsening of Fibrosis 41](#_Toc213429670)

[Supplement S9. Certainty of evidence for each outcome 42](#_Toc213429671)

[Table S9.1: Change in ALT 42](#_Toc213429672)

[Table S9.2: Change in AST 42](#_Toc213429673)

[Table S9.3: Change in LDL 43](#_Toc213429674)

[Table S9.4: Change in HDL 43](#_Toc213429675)

[Table S9.5: Change in Hepatic Fat Fraction (MRI-PDFF) 43](#_Toc213429676)

[Table S9.6: Diarrhea 44](#_Toc213429677)

[Table S9.7: Nausea 44](#_Toc213429678)

[Table S9.8: Fatigue 45](#_Toc213429679)

[Table S9.9: Serious Adverse Events 45](#_Toc213429680)

[Table S9.10: Enhanced Liver Fibrosis (ELF) 45](#_Toc213429681)

[Table S9.11: Adiponectin 46](#_Toc213429682)

[Table S9.12: MASH Resolution with No Worsening of Fibrosis 46](#_Toc213429683)

[Supplement S10. Sensitivity Analysis 47](#_Toc213429684)

[Table S10.1: Leave-One-Out-Analysis for ALT 47](#_Toc213429685)

[Table S10.2: Leave-One-Out-Analysis for AST 47](#_Toc213429686)

[Table S10.3: Leave-One-Out Analysis for LDL 47](#_Toc213429687)

[Table S10.4: Leave-One-Out Analysis for HDL 48](#_Toc213429688)

[Table S10.5: Leave-One-Out Analysis for Change in Hepatic Fat Fraction (MRI-PDFF) 48](#_Toc213429689)

[Table S20.6: Leave-One-Out Analysis for Diarrhea 48](#_Toc213429690)

[Table S10.7: Leave-One-Out Analysis for Nausea 49](#_Toc213429691)

[Table S10.8: Leave-One-Out Analysis for Fatigue 49](#_Toc213429692)

[Table S10.9: Leave-On-Out Analysis for Serious Adverse Events 50](#_Toc213429693)

[Table S10.10: Leave-On-Out Analysis for Enhanced Liver Fibrosis (ELF) 50](#_Toc213429694)

[Table S10.11: Leave-On-Out Analysis for Adiponectin 50](#_Toc213429695)

[Table S10.12: Leave-On-Out Analysis for MASH Resolution with No Worsening of Fibrosis 51](#_Toc213429696)

[Supplement S11. PRISMA checklist 52](#_Toc213429697)

# **Supplement S1. Search strategy**

From inception to January 28^th^, 2025

**PubMed**

(("glucagon receptor agonist" OR "mazdutide" OR "retatrutide" OR "Cotadutide" OR "survodutide") AND ("non-alcoholic fatty liver disease" OR "NAFLD" OR "NASH" OR "liver" OR "steatohepatitis" OR "metabolic-associated steatotic liver disease" OR "MASLD")) OR (("thyroid hormone receptor beta agonist" OR "resmetirom" OR "MGL-3196") AND ("randomized controlled trial" OR "RCT" OR "clinical trial" OR "trial") AND ("liver fat" OR "fibrosis" OR "steatosis" OR "inflammation" OR "histology" OR "weight loss" OR "MRI-PDFF" OR "ALT" OR "AST"))

**Scopus**

(("glucagon receptor agonist" OR "mazdutide" OR "retatrutide" OR "Cotadutide" OR "survodutide") AND ("non-alcoholic fatty liver disease" OR "NAFLD" OR "NASH" OR "liver" OR "steatohepatitis" OR "metabolic-associated steatotic liver disease" OR "MASLD")) OR (("thyroid hormone receptor beta agonist" OR "resmetirom" OR "MGL-3196") AND ("randomized controlled trial" OR "RCT" OR "clinical trial" OR "trial") AND ("liver fat" OR "fibrosis" OR "steatosis" OR "inflammation" OR "histology" OR "weight loss" OR "MRI-PDFF" OR "ALT" OR "AST"))

**Cochrane**

(("glucagon receptor agonist" OR "mazdutide" OR "retatrutide" OR "Cotadutide" OR "survodutide") AND ("non-alcoholic fatty liver disease" OR "NAFLD" OR "NASH" OR "liver" OR "steatohepatitis" OR "metabolic-associated steatotic liver disease" OR "MASLD")) OR (("thyroid hormone receptor beta agonist" OR "resmetirom" OR "MGL-3196") AND ("randomized controlled trial" OR "RCT" OR "clinical trial" OR "trial") AND ("liver fat" OR "fibrosis" OR "steatosis" OR "inflammation" OR "histology" OR "weight loss" OR "MRI-PDFF" OR "ALT" OR "AST"))

**ClinicalTrials.gov**

(("glucagon receptor agonist" OR "mazdutide" OR "retatrutide" OR "Cotadutide" OR "survodutide") AND ("non-alcoholic fatty liver disease" OR "NAFLD" OR "NASH" OR "liver" OR "steatohepatitis" OR "metabolic-associated steatotic liver disease" OR "MASLD")) OR (("thyroid hormone receptor beta agonist" OR "resmetirom" OR "MGL-3196") AND ("randomized controlled trial" OR "RCT" OR "clinical trial" OR "trial") AND ("liver fat" OR "fibrosis" OR "steatosis" OR "inflammation" OR "histology" OR "weight loss" OR "MRI-PDFF" OR "ALT" OR "AST"))

# **Supplement S2. Network plots of treatment comparisons**

## Figure S2.1: Network Plot of Treatment Comparisons for Changes in ALT


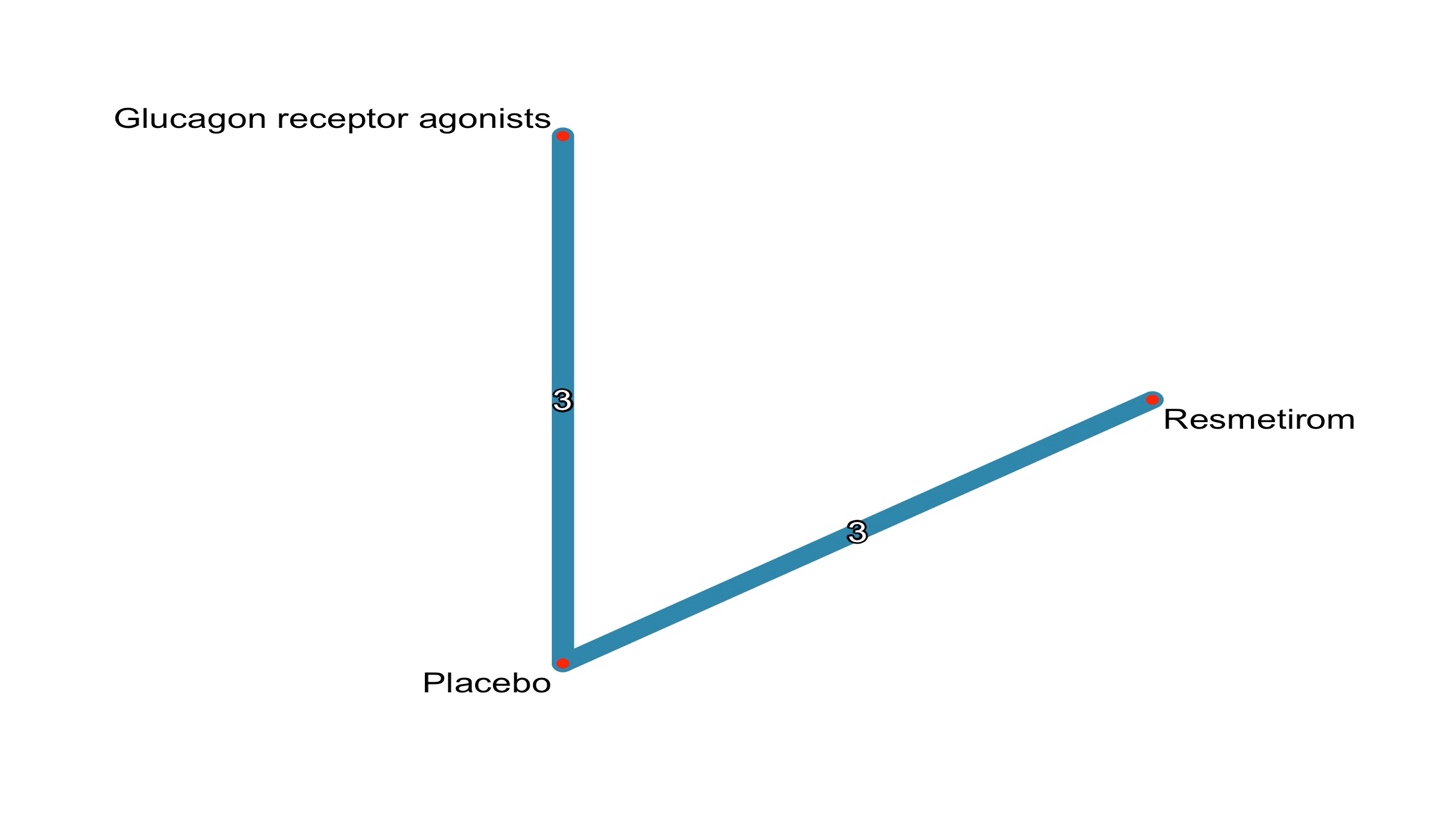


## Figure S2.2: Network Plot of Treatment Comparisons for Changes in AST


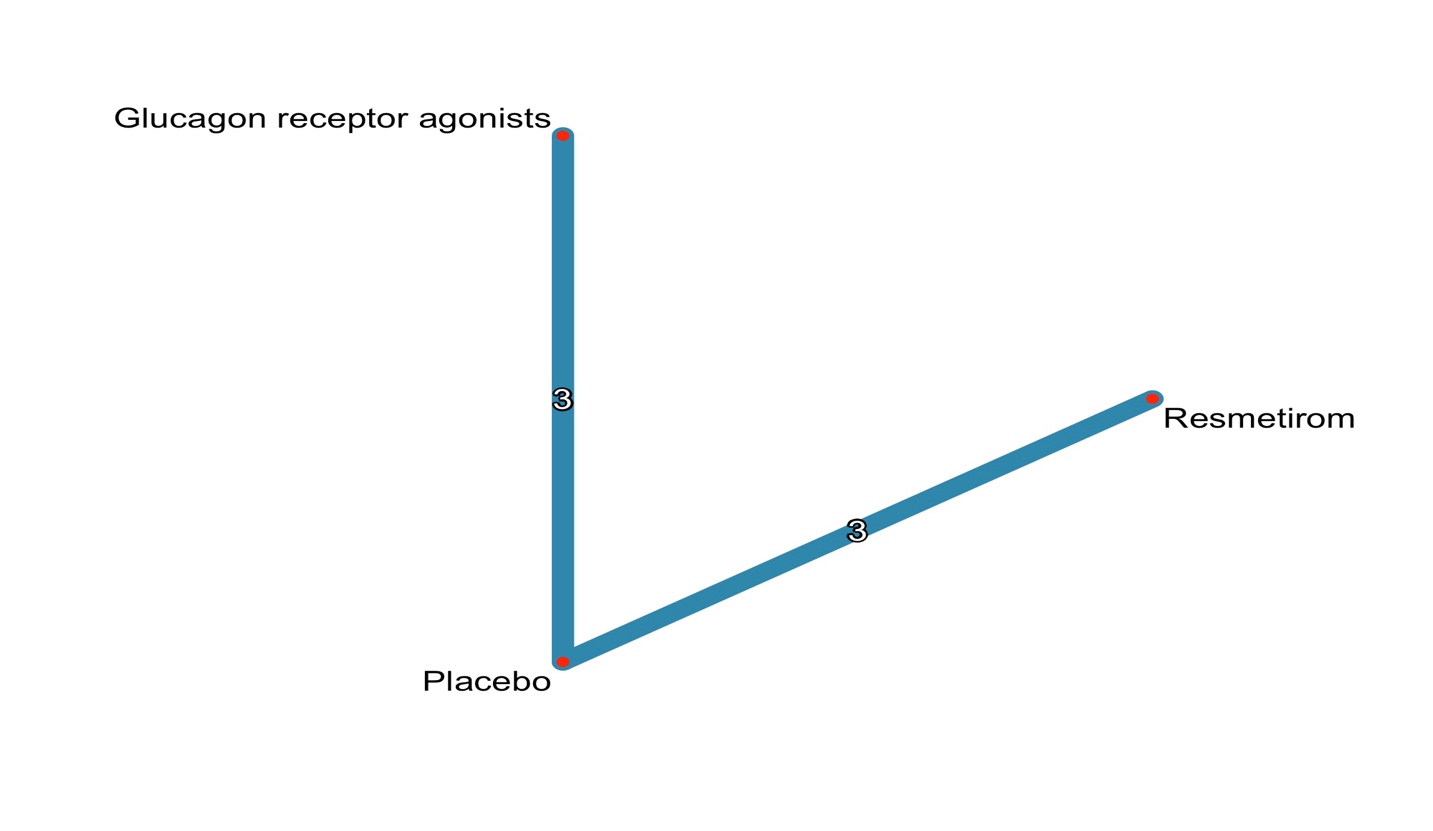


## Figure S2.3: Network Plot of Treatment Comparisons for Changes in LDL


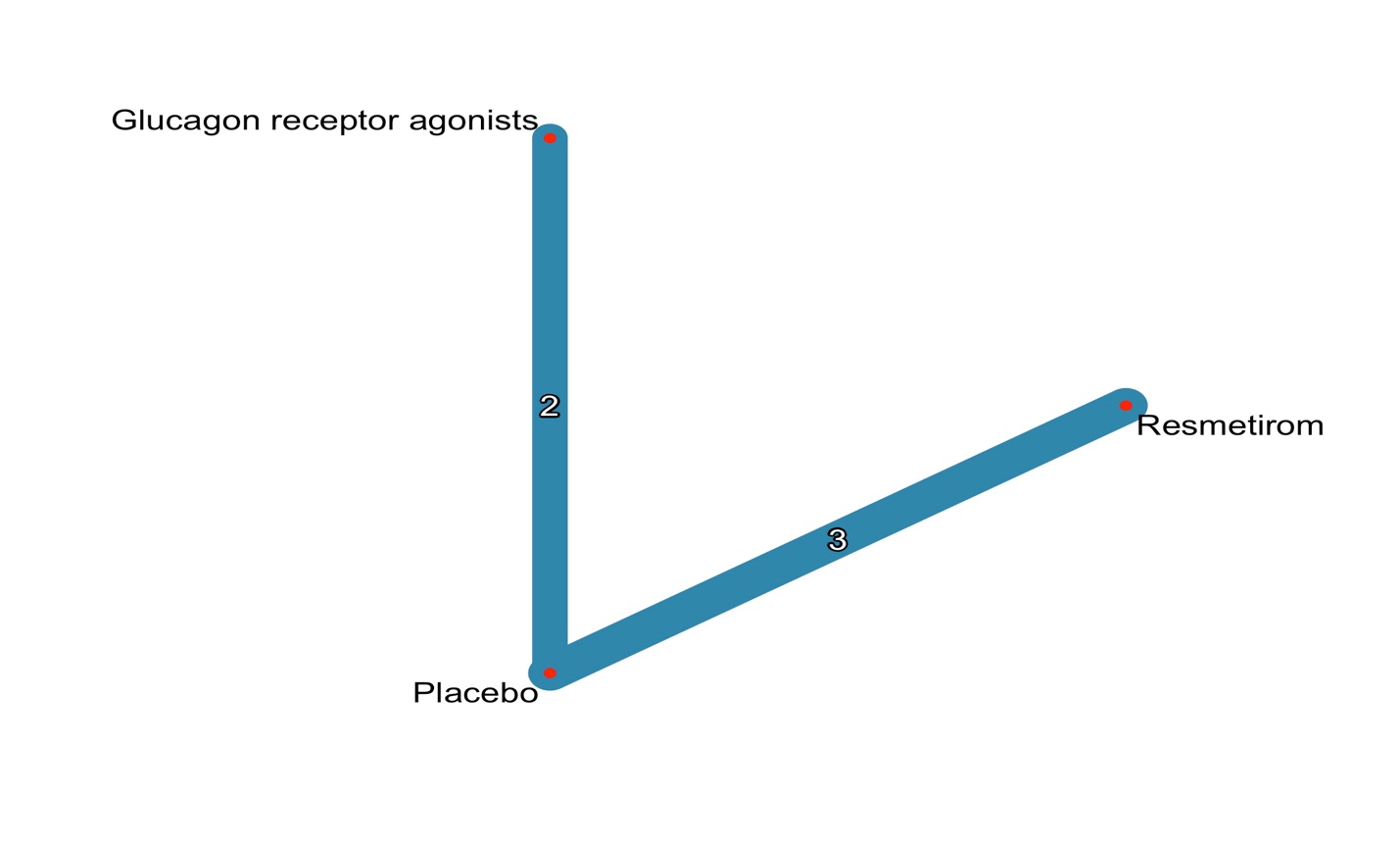


## Figure S2.4: Network Plot of Treatment Comparisons for Changes in HDL


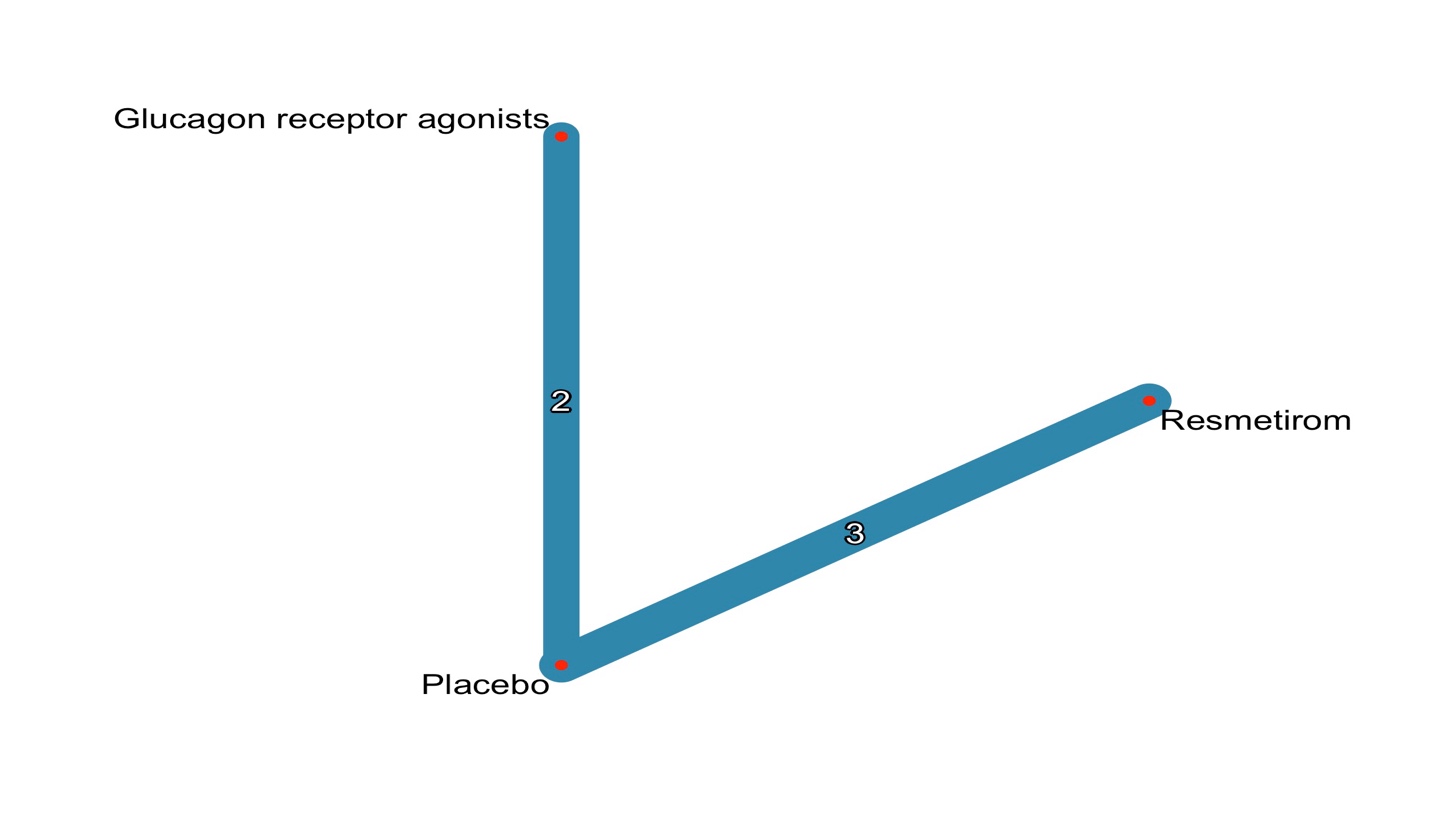


## Figure S2.5: Network Plot of Treatment Comparisons for Hepatic Fat Reduction (MRI-PDFF)


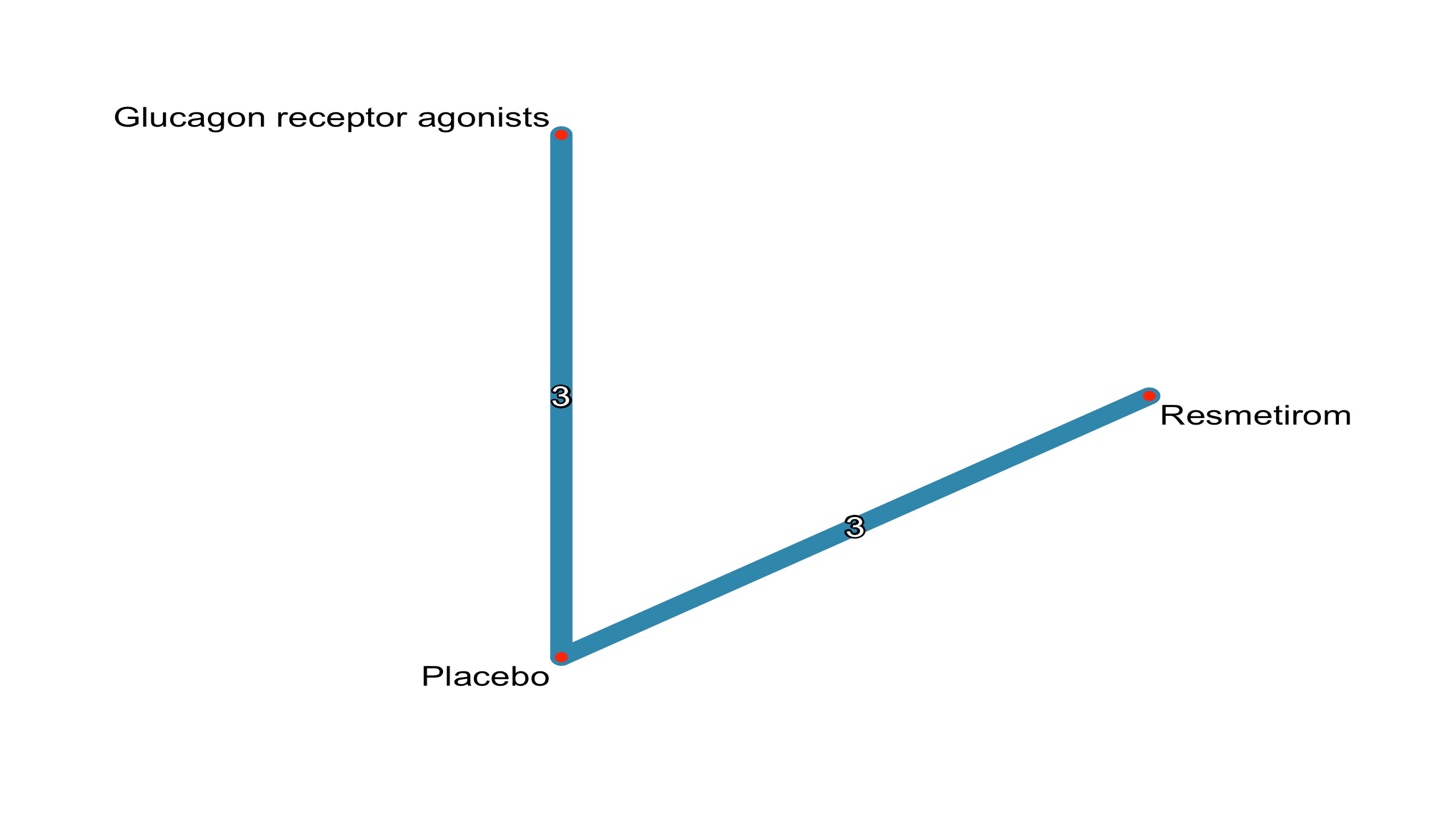


## Figure S2.6: Network Plot of Treatment Comparisons for Diarrhea


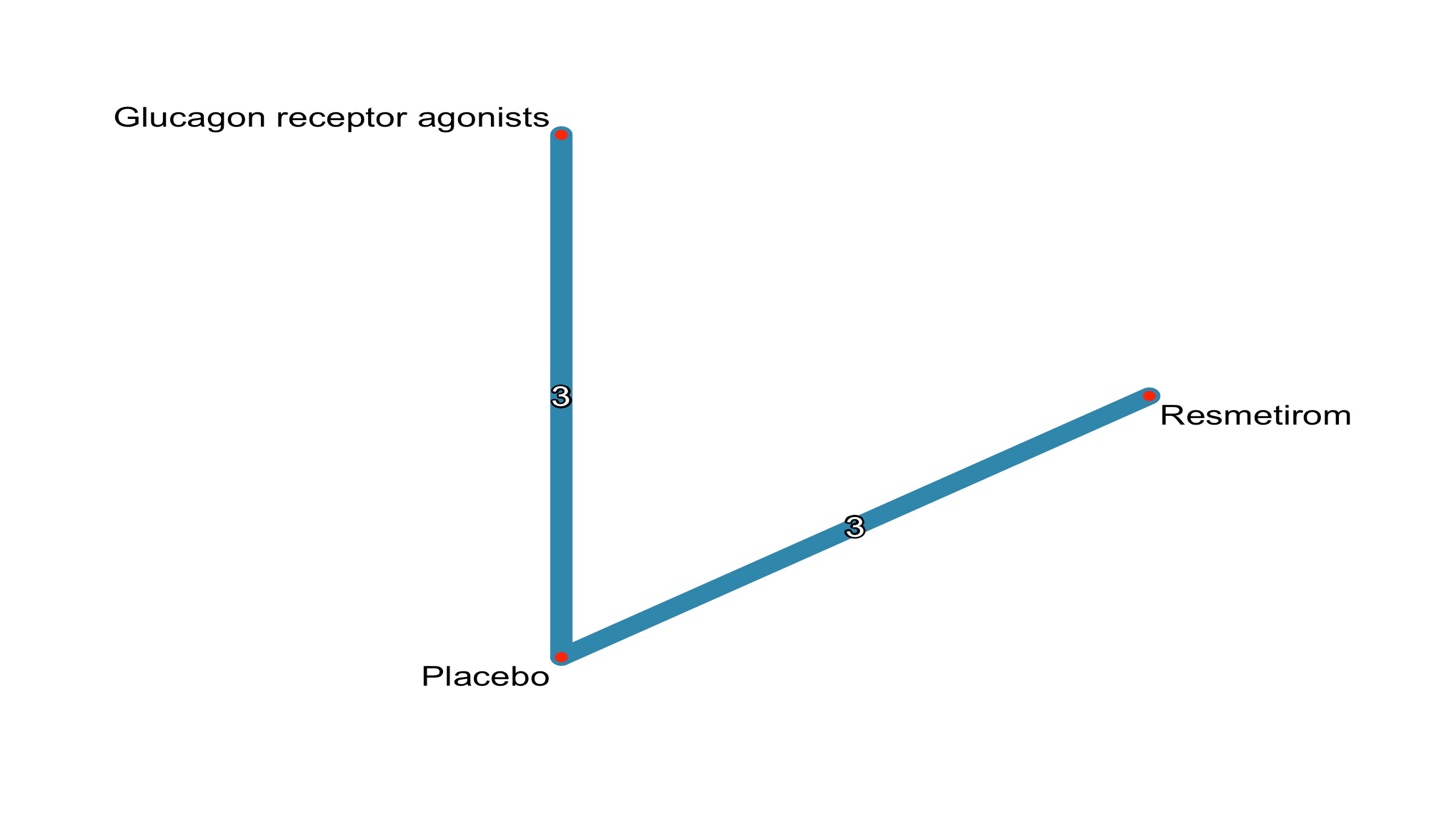


## Figure S2.7: Network Plot of Treatment Comparisons for Nausea


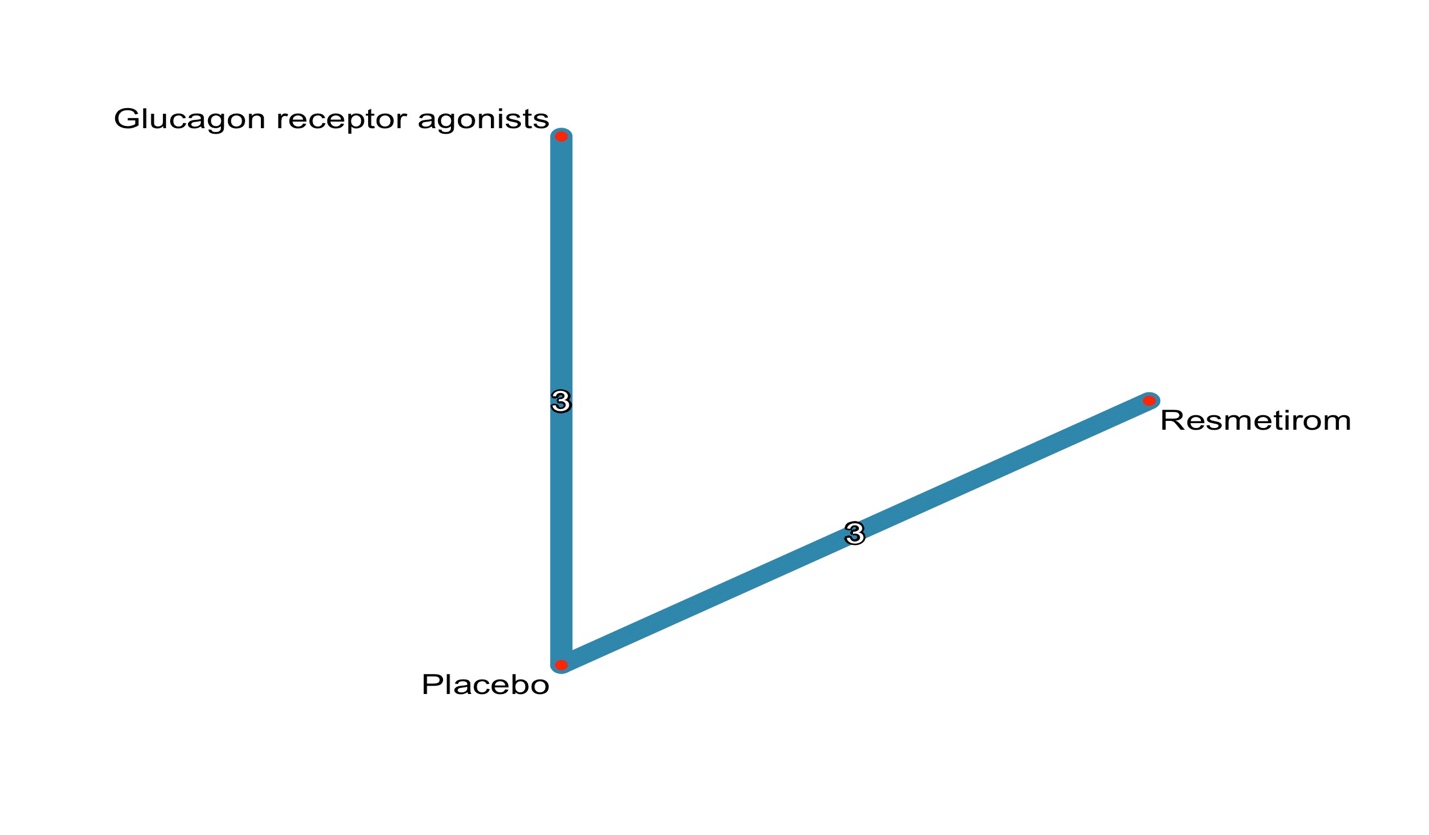


## Figure S2.8: Network Plot of Treatment Comparisons for Fatigue


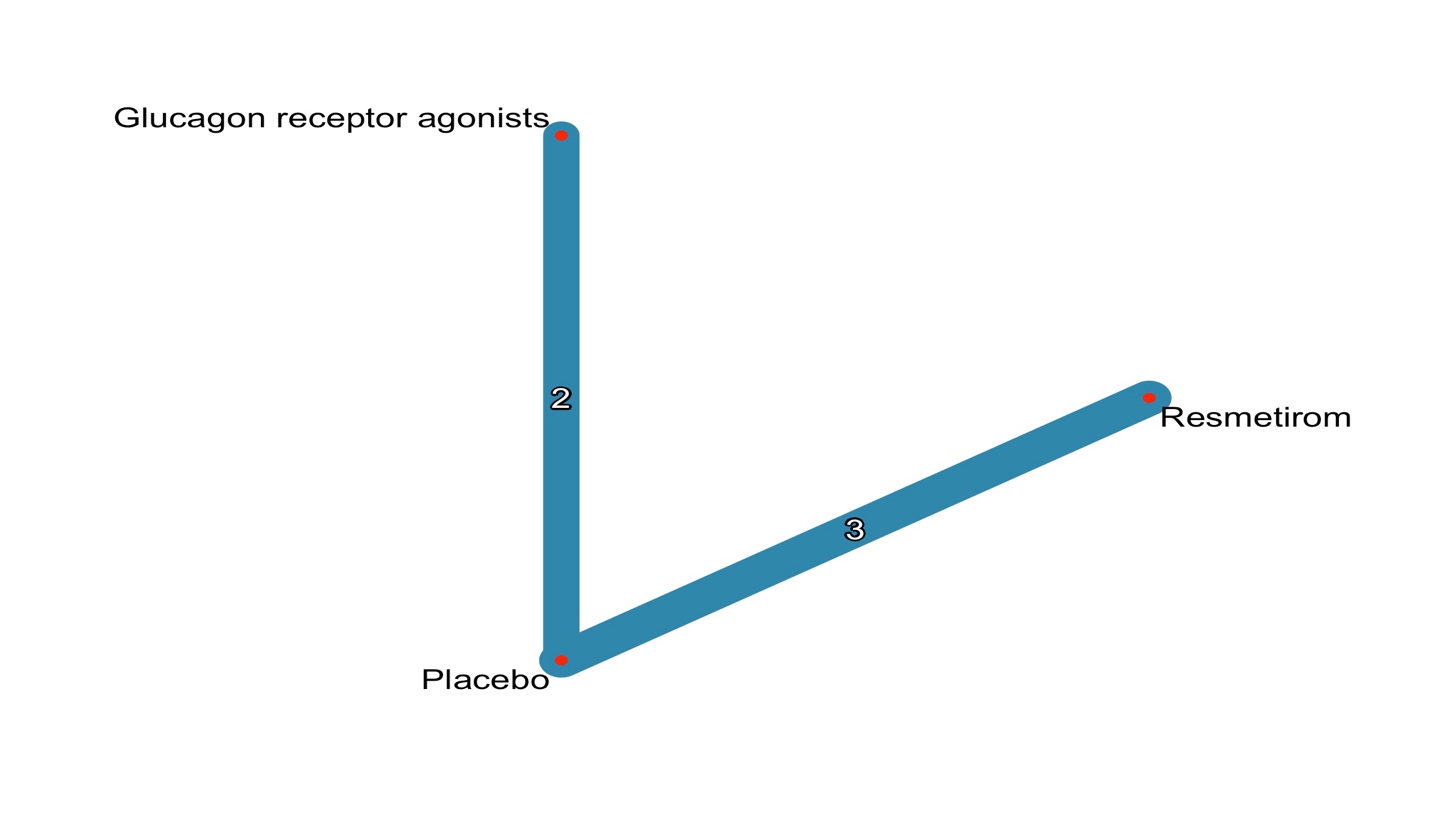


## Figure S2.9: Network Plot of Treatment Comparisons for Serious Adverse Events


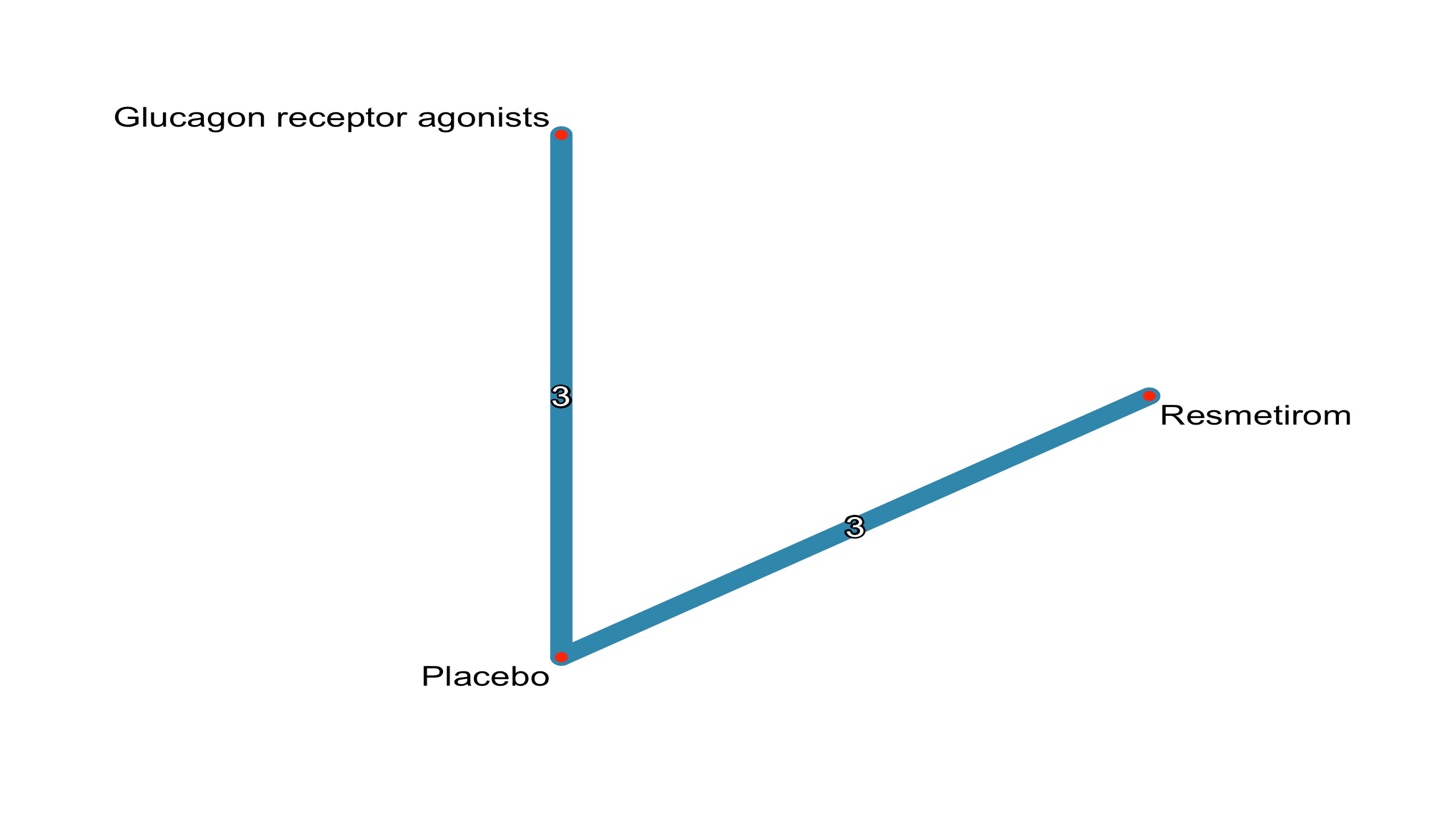


## Figure S2.10: Network Plot of Enhanced Liver Fibrosis (ELF)


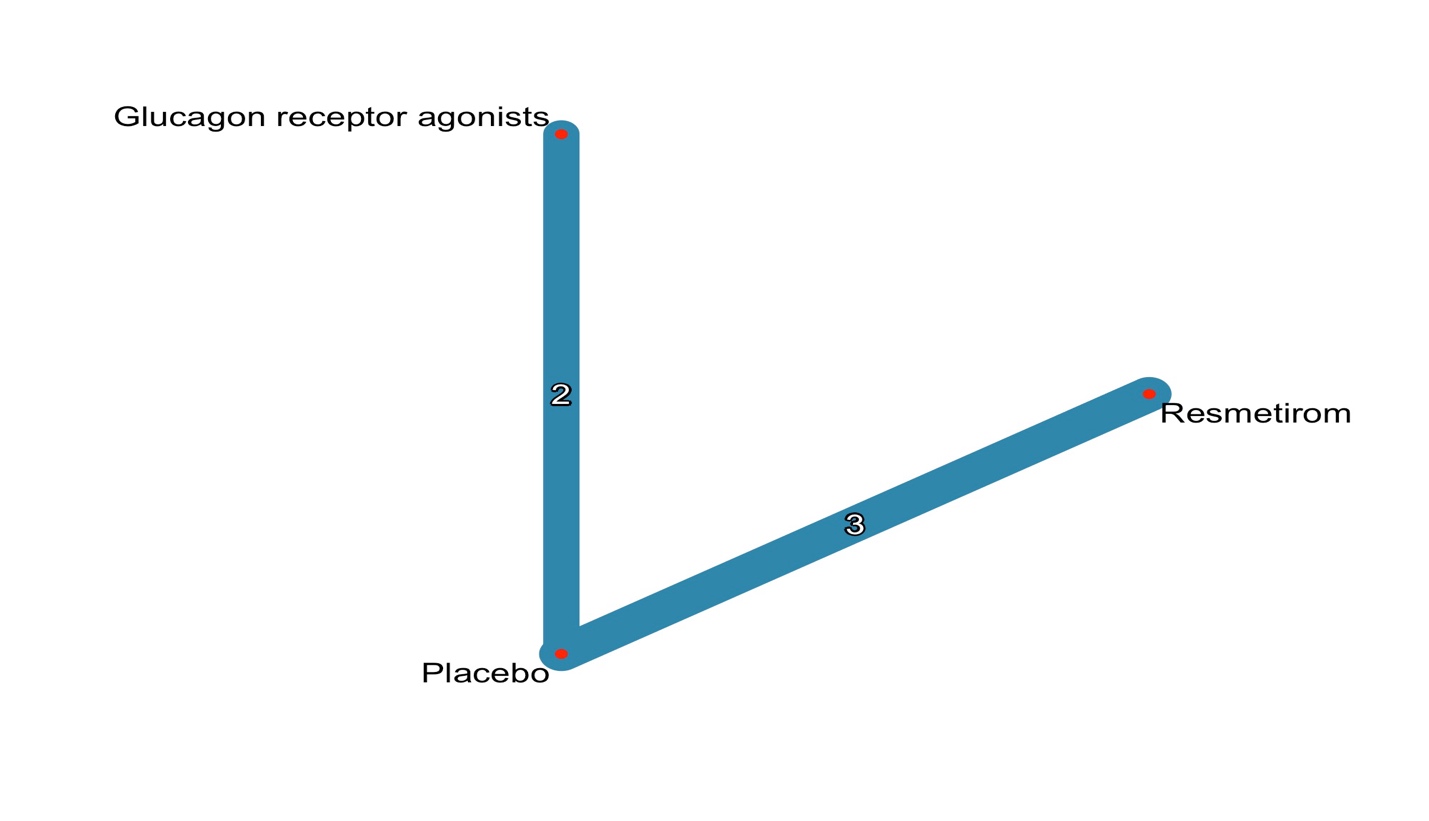


## Figure S2.11: Network Plot of Adiponectin


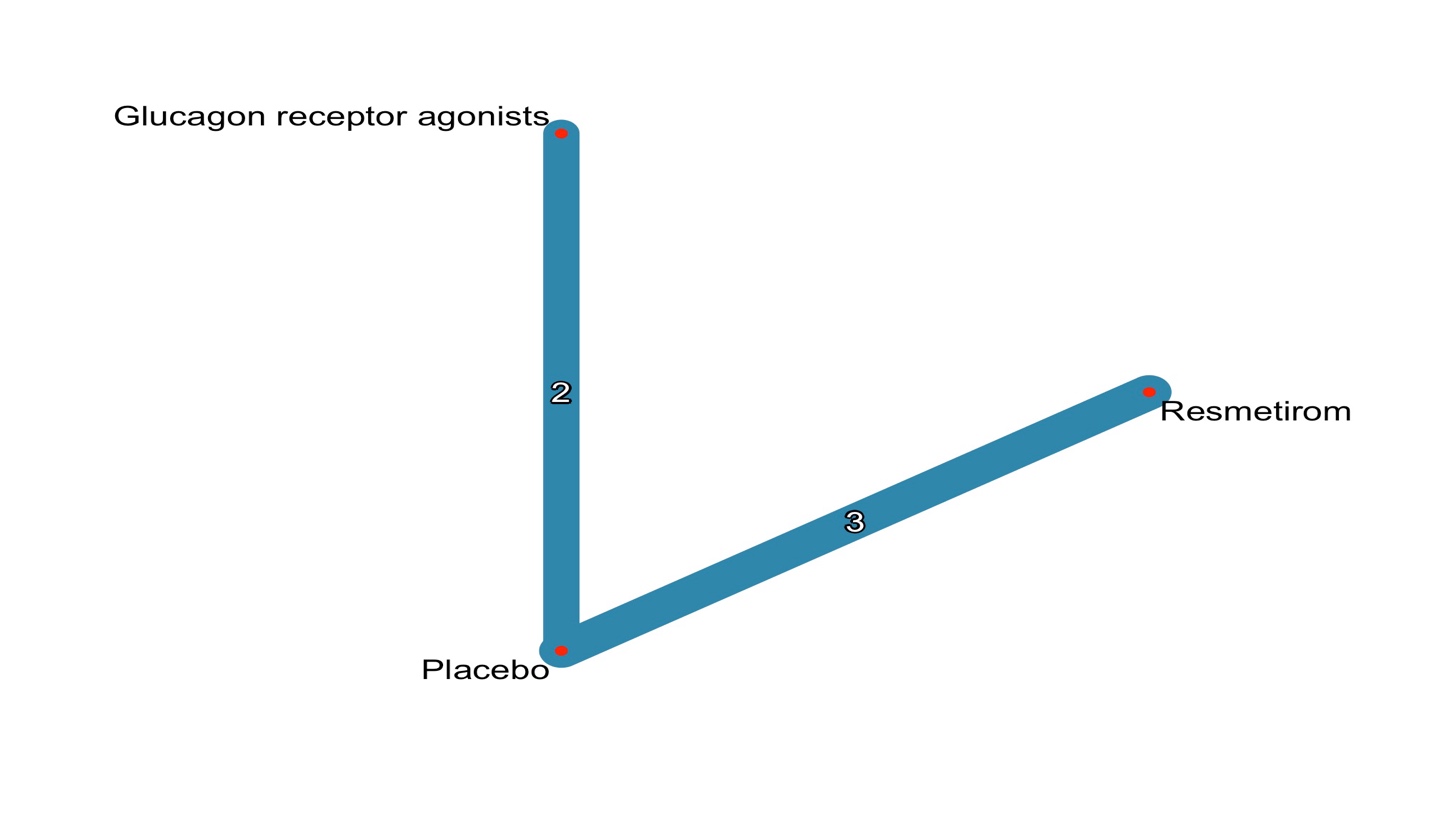


## Figure S2.12: Network Plot of MASH Resolution with No Worsening of Fibrosis


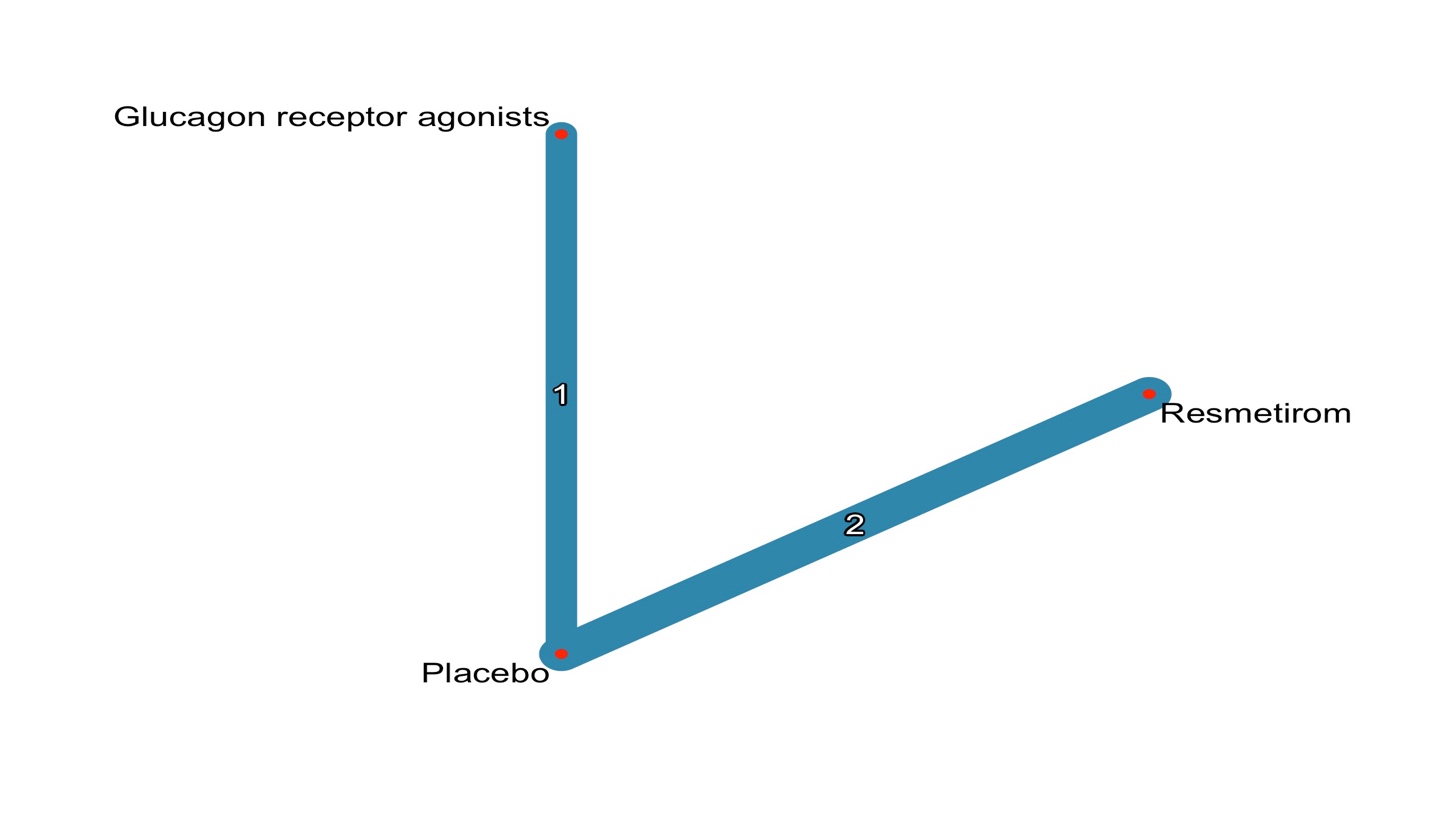


# **Supplement S3 Baseline characteristics of the included studies**

The table summerizes various clinical trials, detailing study design, registration, duration, treatment arms, and population characteristics.

| **Study ID** | **Trial Phase** | **Year** | **NCT Code** | **Population Included** | **Population Excluded** | **Number of Participants** | **Intervention** | **Comparator** | **Duration (weeks)** |
| --- | --- | --- | --- | --- | --- | --- | --- | --- | --- |
| Harrison,2019 | Phase 2 | 2019 | NCT02912260 | Adults (≥18) with biopsy-confirmed NASH or strong NASH indicators (metabolic syndrome + imaging or past biopsy), ≥9–10% liver fat on MRI-PDFF, and liver biopsy showing stage 1–3 fibrosis with NAFLD score ≥4 (≥1 in steatosis, ballooning, inflammation). | Cirrhosis or advanced liver disease beyond stage 3 fibrosis, uncontrolled medical conditions or contraindications to the study treatment, use of specific medications or interventions that could interfere with the study's outcomes. | 125 | Resmetirom 80 mg | Placebo | 36 |
| Harrison,2023 | Phase 3 | 2023 | NCT04197479 | Adults ≥18. Women: non-reproductive or using strict birth control; men: sterile or using contraception, no sperm donation for 30 days. Liver fat/fibrosis confirmed by FibroScan, MRE, MRI-PDFF, or recent biopsy. MRI-PDFF ≥8% or biopsy/FibroScan criteria. Stable lipid therapy ≥30 days. eGFR ≥45. | ≥2 drinks/day (men), ≥1.5 (women); active thyroid disease; advanced liver disease (F4, MELD ≥12, ALT >250, HCC, decompensation); recent autoimmune treatment; cancer <5 yrs; serious conditions (EF <30%, NYHA III/IV, MI/stroke <12 wks, prolonged QTcF, uncontrolled BP/arrhythmia); bariatric surgery <5 yrs; unstable GLP-1, vit E >400 IU, pioglitazone >15 mg (unless stable); NAFLD-related drugs; weight change >5% (12 wks); HbA1c >9%; platelets <140K (unless Fib-4 <3.5); investigational drugs/surgery <60 days/6 wks; IV drug use <5 yrs; noncompliance risk; resmetirom allergy; immunocompromised. | 972 | Resmetirom 80 mg  Resmetirom 100 mg | Placebo | 52 |
| Harrison,2024 | Phase 3 | 2024 | NCT03900429 | Adults ≥18 with ≥3 metabolic risk factors. VCTE: CAP ≥280 dB/m & stiffness ≥8.5 kPa or recent biopsy confirming NASH. Biopsy: NAS ≥4 (≥1 in steatosis, inflammation, ballooning); ≥50% with F3 fibrosis. Stable weight (<5% change in 3 months) and GLP-1 dose (≥6 months). | Alcohol consumption >20 g/day for women or >30 g/day for men. HbA1c >9.0% at screening.  Causes of chronic liver disease other than noncirrhotic NASH. | 966 | Resmetirom 80 mg  Resmetirom 100 mg | Placebo | 52 |
| Shankar,2024 | Phase 2 | 2024 | NCT04019561 | Adults ≥18, BMI ≥30, HbA1c ≤9.5%; if T2DM, managed with stable diet or select meds (≥3 months). Biopsy-confirmed MASLD/MASH (NAS ≥4 with ≥1 in all components) within 6 months, fibrosis stage F1–F3 (F1 ≤25%). MRI-PDFF ≥10% liver fat. Women of childbearing potential: effective contraception, negative pregnancy test, not breastfeeding. | Excludes other liver diseases, cirrhosis, transplant, alcohol/substance abuse, insulin use, severe GI/CV issues, recent weight loss, cancer (except treated skin/cervical), mental illness, recent NAFLD/MASH therapies, high-dose vitamin E/GLP-1 (≤3 months), recent trial use (<30 days), allergies, pancreatitis, uncontrolled BP, abnormal labs, MRI contraindications, HIV+, thyroid cancer risk, and pregnancy/breastfeeding. | 74 | Cotadutide 300 ug  Cotadutide 600 ug | Placebo | 19 |
| Sanyal,2024_a | Phase 2 | 2024 | NCT04881760 | Age 18–75, BMI ≥30–50 or 27–<30 with weight-related comorbidities. Able/willing to self-inject or receive injections and follow study procedures. Liver fat ≥10% by MRI-PDFF (subset). Male or female; contraception required if of childbearing potential. Informed consent given.. | No diabetes, recent major weight change, obesity surgery, or weight-altering drugs; limited alcohol; no recent marijuana; no recent serious heart events; no active cancer (5 yrs); controlled BP; only NAFLD liver disease; eGFR ≥45; no pancreatitis, gallbladder disease, HIV; no major mental illness (2 yrs); no MEN2/medullary thyroid cancer history; women must use contraception and not be pregnant/breastfeeding/planning pregnancy. | 98 | Retatrutide 1mg  Retatrutide 4 mg  Retatrutide 8 mg  Retatrutide 12 mg | Placebo | 48 |
| Sanyal,2024_b | Phase 2 | 2024 | NCT04771273 | Adults 18–80 with biopsy-confirmed MASH fibrosis (F1–F3) ≤6 months or at screening, liver fat ≥8%, stiffness >6 kPa. BMI ≥25, weight ≥70 kg. Consent provided. Women of childbearing potential must use two effective contraceptives, one highly effective (<1% failure). | Excludes recent heavy alcohol use; liver-toxic meds; liver disease, cancer, transplant; thyroid cancer/neoplasia; pancreatitis/high enzymes/triglycerides; HIV, TB, COVID; abnormal labs; serious organ disease; recent suicide/depression; bariatric surgery (except stable band); high HR/BP; heart issues; recent MI/stroke/bypass; pregnancy/breastfeeding; MRI issues; recent trial; unsafe conditions. | 293 | survodutide 2.4 mg  survodutide 4.8 mg  survodutide 6.0 mg | Placebo | 48 |

# **Supplement S4. Baseline characteristics of the participants**

The table summarizes the baseline characteristics of patients included in the study. Characteristics include age, male, female, body weight, BMI, ALT, AST, MRI- proton density fat fraction, diabetes, HbA1c, waist circumference, HDL and LDL.

| **Study ID** | **Age (mean, SD)** | **Male (%)** | **Female (%)** | **Body weight, kg (mean, SD)** | **BMI (kg/m²)** | **ALT (IU/L)** | **AST (IU/L)** | **MRI-proton density fat fraction, %**  **fat fraction (SD)** | **Diabetes (%)** | **HbA1c**  **(%)** | **Waist circumference, cm (mean, SD)** | **HDL, mg/dl (mean, SD)** | **LDL, mg/dl (mean, SD)** | **Enhanced Liver Fibrosis (ELF) U/A (mean, SD)** | **Adiponectin mg/L (mean, SD)** |
| --- | --- | --- | --- | --- | --- | --- | --- | --- | --- | --- | --- | --- | --- | --- | --- |
| **Harrison,2019** | 50.4 ± 9.64 | 49.6% | 50.4% | 99.85 ± 21.65 | 35.07 ± 6.14 | 53.31 ± 31.05 | 36.05 ± 18.71 | 20.0 ± 6.7 | 28.5 ± 41.4 | 6.3 ± 1.1 | 110.04 ± 18.46 | 44.26 ± 12.73 | 113.14 ± 30.23 | 9.2 ± 0.93 | 4.92 ± 2.73 |
| **Harrison,2023** | 56.1 ± 11.8 | 45.6% | 54.4% | NR | 35.3 ± 6.04 | 37.1 ± 26.7 | 25.5 ± 14.2 | 17.9 ± 7.0 | 158.1 ± 49.0 | NR | NR | 43.7 ± 13.82 | 109.1 ± 37.14 | 10.26 ± 0.40 | 4.6 ± 2.7 |
| **Harrison,2024** | 56.7 ± 11.77 | 43.9% | 56.1% | 100.6 ± 22.7 | 35.74 ± 6.78 | 54.7 ± 32.3 | 40.5 ± 23.2 | 16.7 ± 6.8 | 215.2 ± 67.3 | NR | NR | NR | 105.3 ± 38.3 | 9.7 ± 0.9 | 4.07 ± 2.47 |
| **Shankar,2024** | 55.8 ± 11.65 | 44.6% | 55.4% | 100.1 ± 18.67 | 37.27 ± 5.83 | 46.1 ± 30.68 | 36.2 ± 19.3 | 19.4 ± 7.8 | 13.7 ± 55.0 | 6.7 ± 1.3 | NR | 21.0 ± 5.39 | 50.9 ± 16.15 | NR | NR |
| **Sanyal,2024_a** | 46.6 ± 12 | 53.1% | 46.9% | 110.2 ± 18.6 | 38.4 ± 5.2 | 32.86 ± 3.83 | 24.2 ± 1.71 | 19.1 ± 6.9 | NR | 5.6 ± 0.4 | 118.3 ± 13.4 | NR | NR | 8.1 ± 0.21 | 3.99 ± 0.55 |
| **Sanyal,2024_b** | 50.8 ± 12.8 | 47% | 53% | 100.8 ± 22.37 | 35.81 ± 6.41 | 57.8 ± 41.8 | 47.3 ± 36.5 | 19.6 ± 7.5 | 113 ± 39 | 7.0 ± 1.0 | 113.81 ± 13.91 | 20.35 ± 5.37 | 11.96 ± 17.12 | NR | NR |

## **Abbreviations**:

**BMI:** Body Mass Index **HDL:** High density lipoprotein

**ALT:** Alanine aminotransferase **LDL:** Low density lipoprotein

**AST:** Aspartate aminotransferase

**HbA1c:** Glycated hemoglobin

# **Supplement S5. Risk of bias table**

| Study ID | Random Sequence Generation | Allocation Concealment | Blinding of Participants/Personnel | Blinding of Outcome Assessment | Incomplete Outcome Data | Selective Reporting | Other Bias | Overall Judgment |
| --- | --- | --- | --- | --- | --- | --- | --- | --- |
| Harrison,2019 | Low risk | Low risk | Low risk | Low risk | Some concerns | Low risk | Some concerns | Some concerns |
| Harrison,2023 | Low risk | Low risk | Low risk | Low risk | Some concerns | Low risk | Some concerns | Some concerns |
| Harrison,2024 | Low risk | Low risk | Low risk | Low risk | Some concerns | Low risk | Some concerns | Some concerns |
| Shankar,2024 | Low risk | Low risk | Low risk | Low risk | Low risk | Low risk | Some concerns | Some concerns |
| Sanyal,2024_a | Low risk | Low risk | Low risk | Some concerns | Some concerns | Low risk | Some concerns | Some concerns |
| Sanyal,2024_b | Low risk | Low risk | Low risk | Low risk | Some concerns | Low risk | Some concerns | Some concerns |

# **Supplement S6. Publication bias (funnel plot)**

## Figure S6.1: Change in ALT


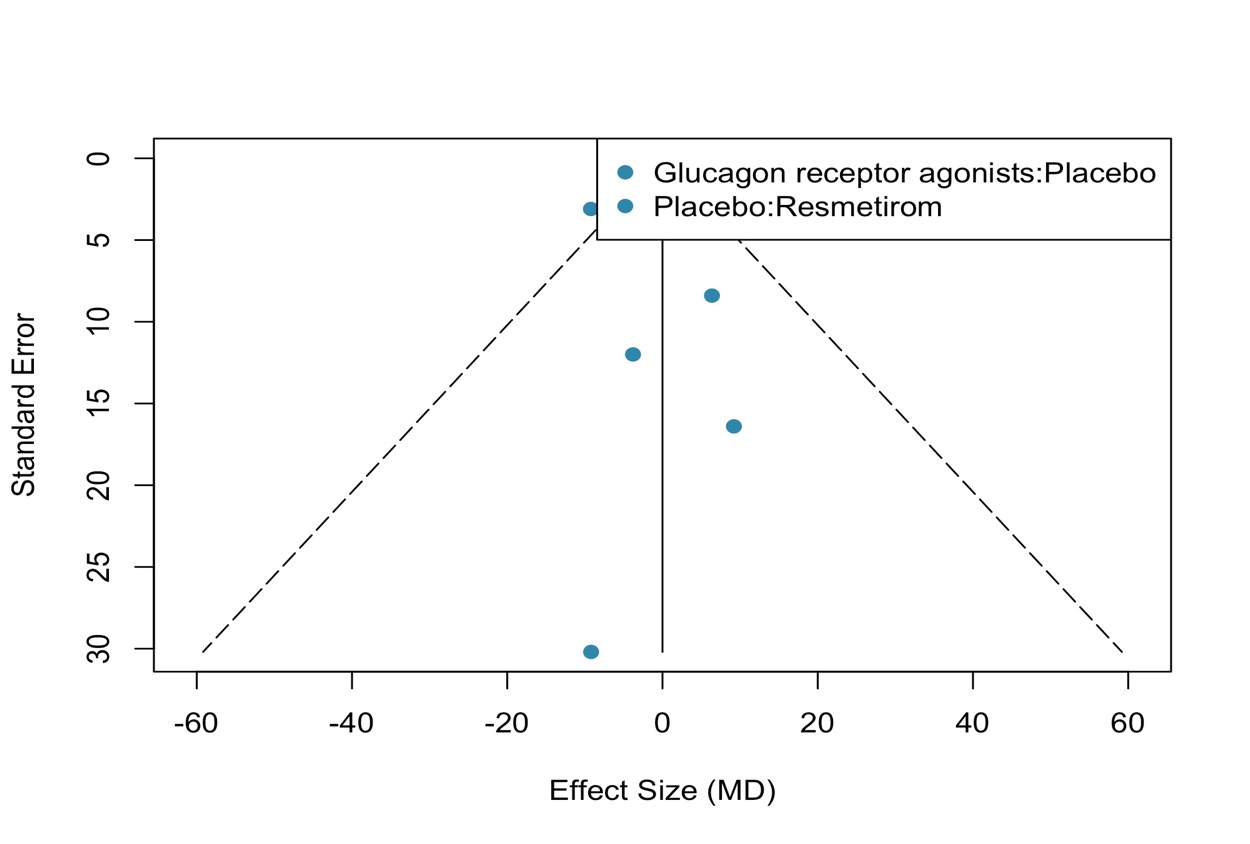


## Figure S6.2: Change in AST


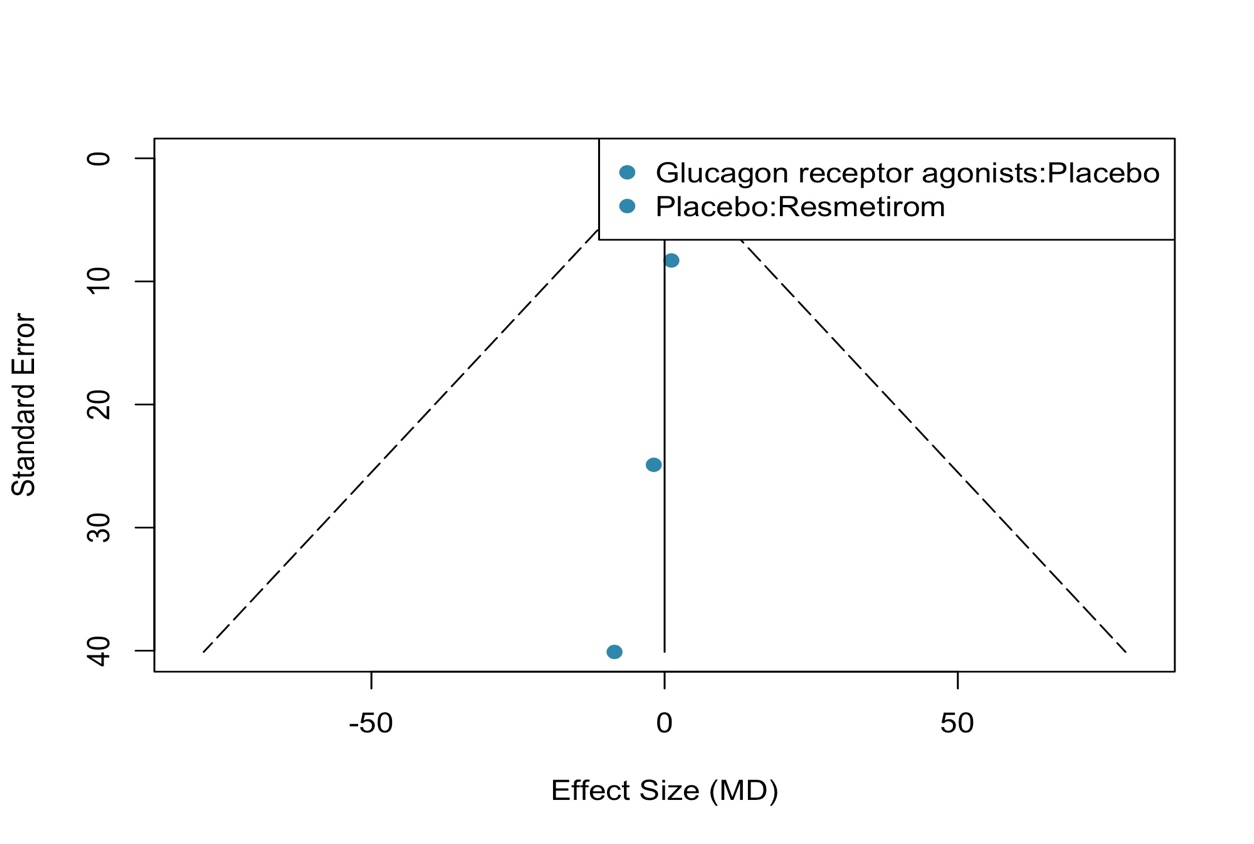


## Figure S6.3: Change in LDL


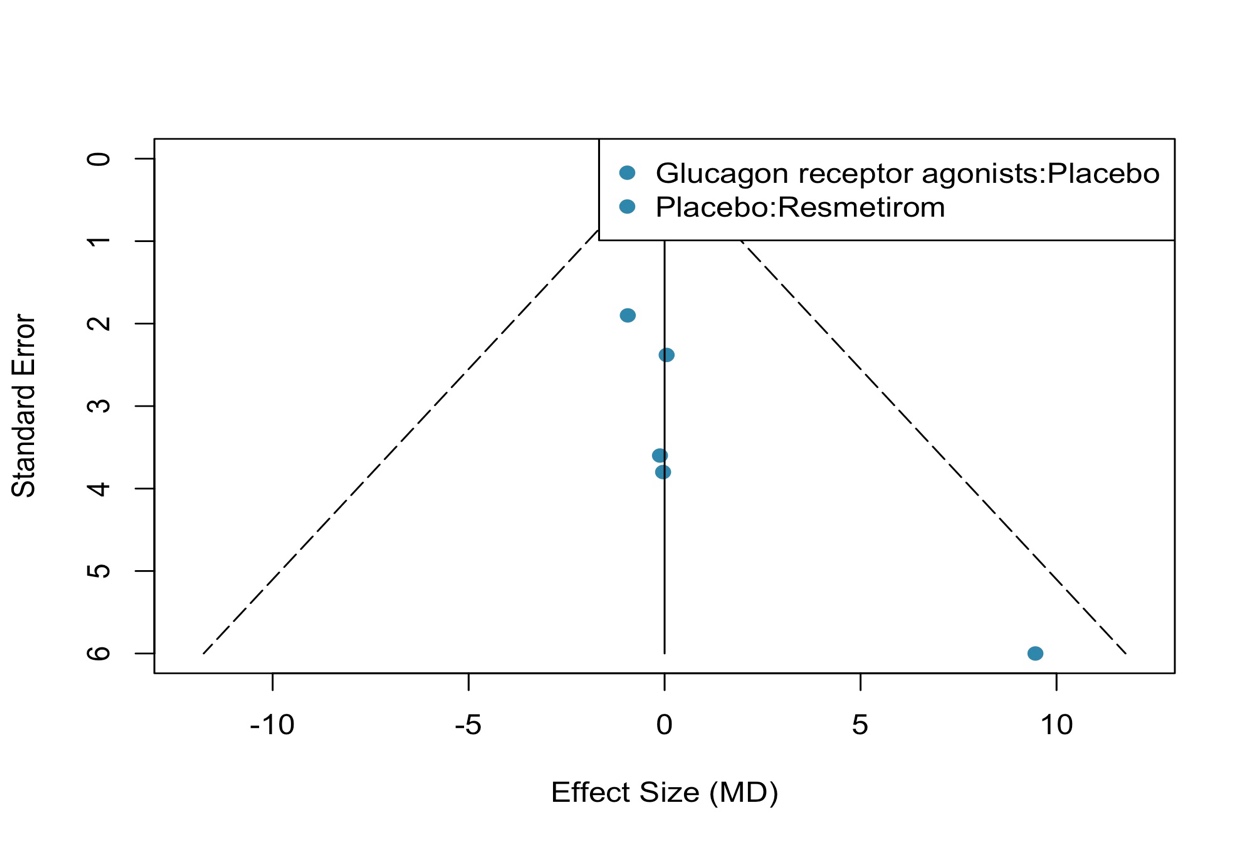


## Figure S6.4: Change in HDL


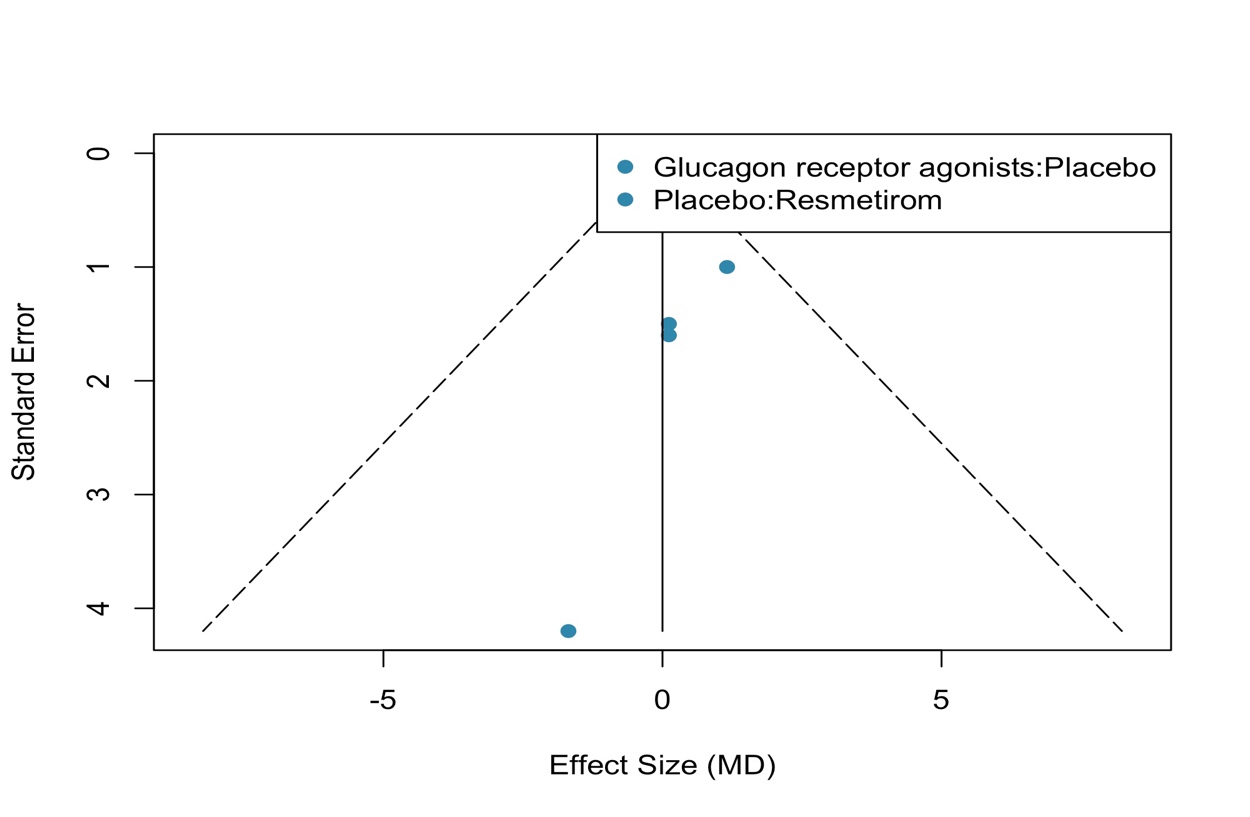


## Figure S6.5: Hepatic Fact Reduction (MRI-PDFF)


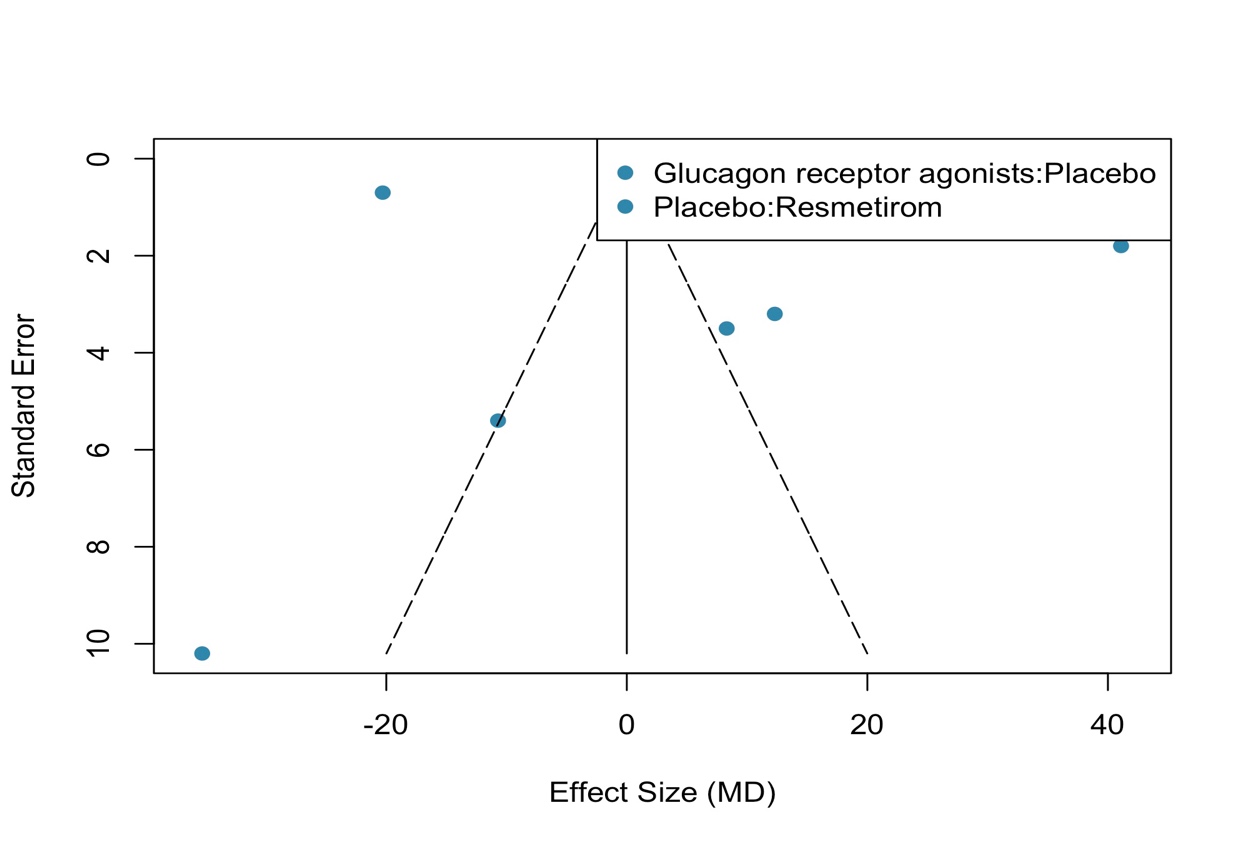


## Figure S6.6: Diarrhea


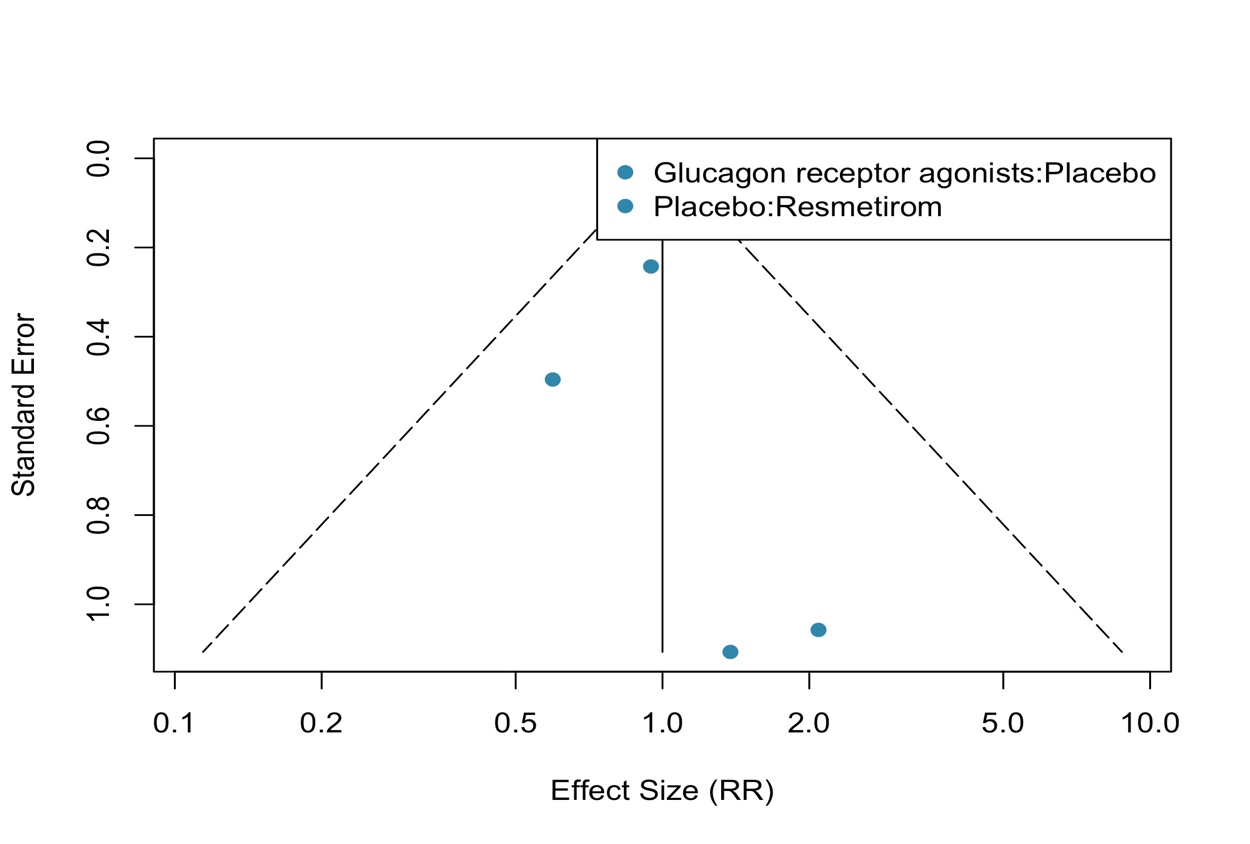


## Figure S6.7: Nausea


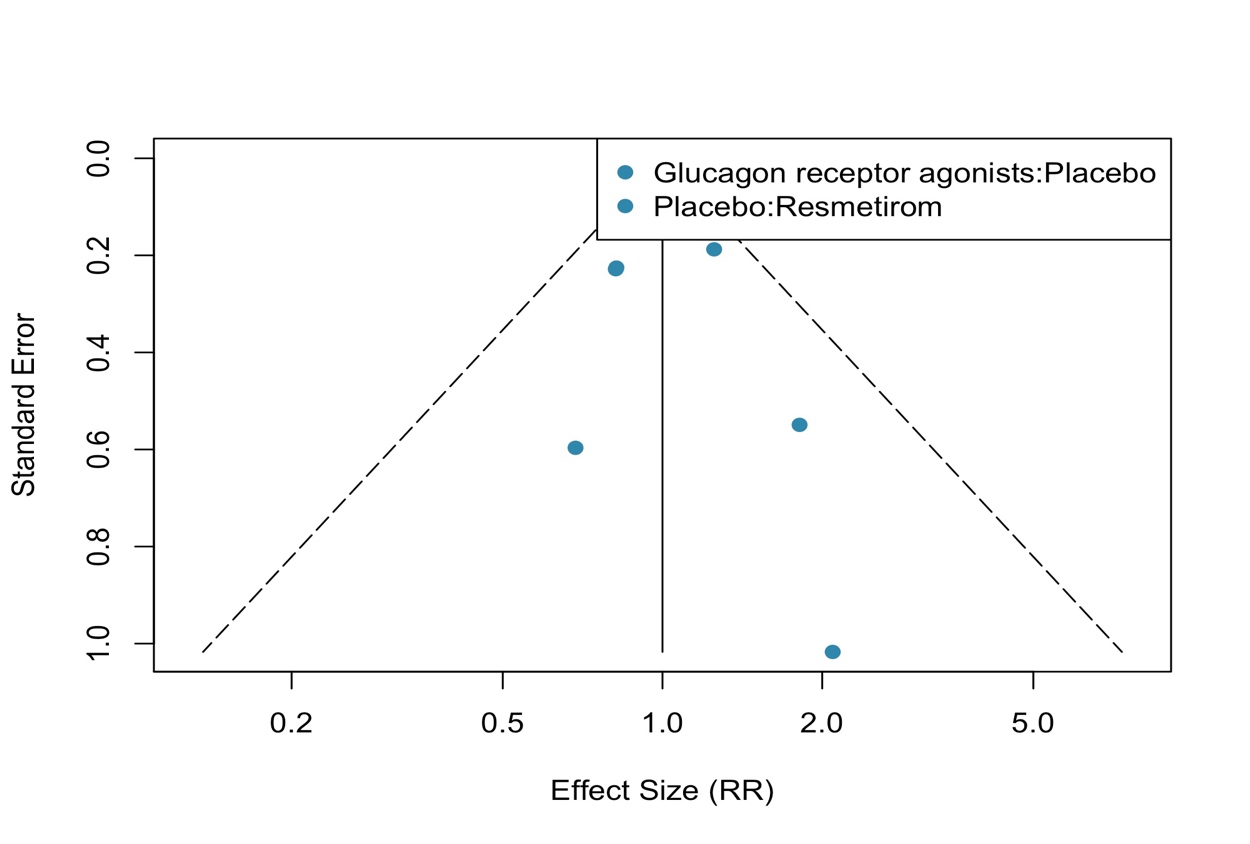


## Figure S6.8: Fatigue


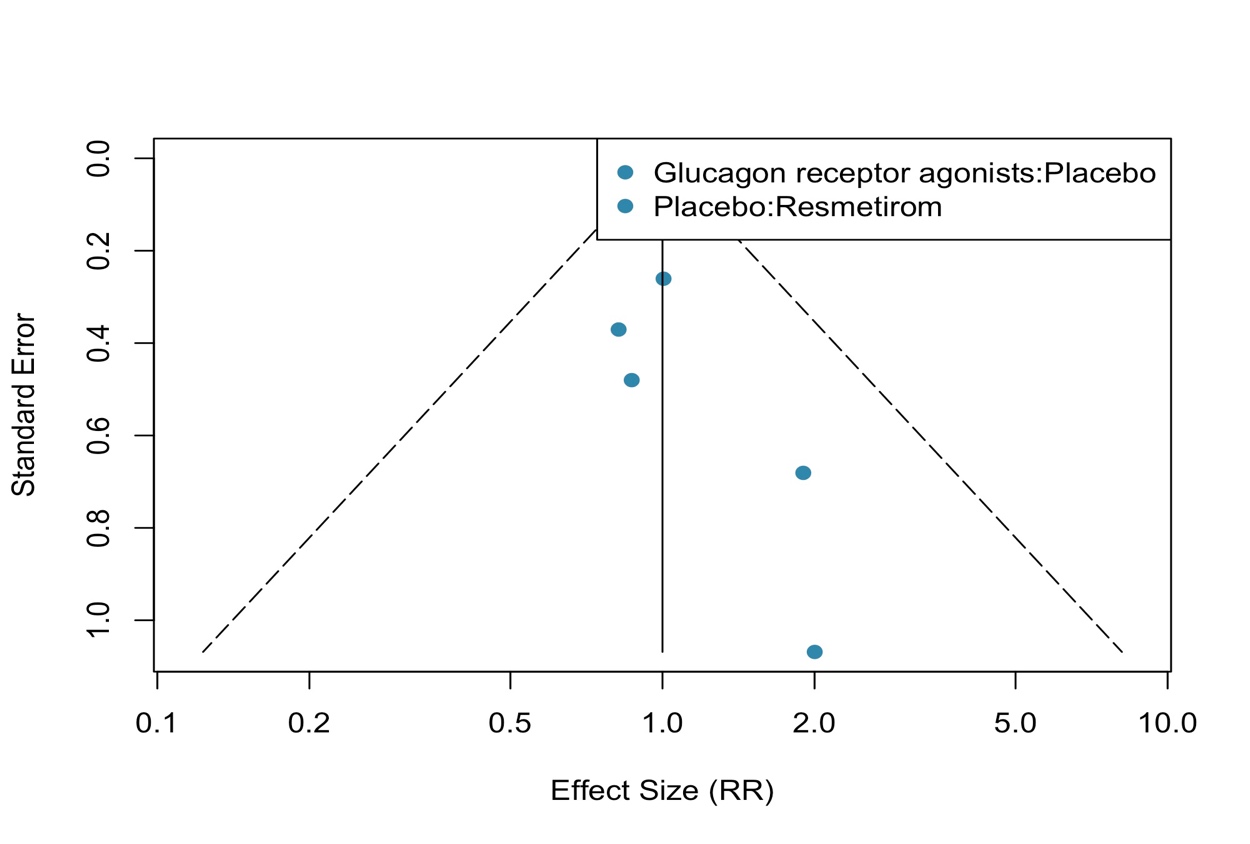


## Figure S6.9: Serious Adverse Events


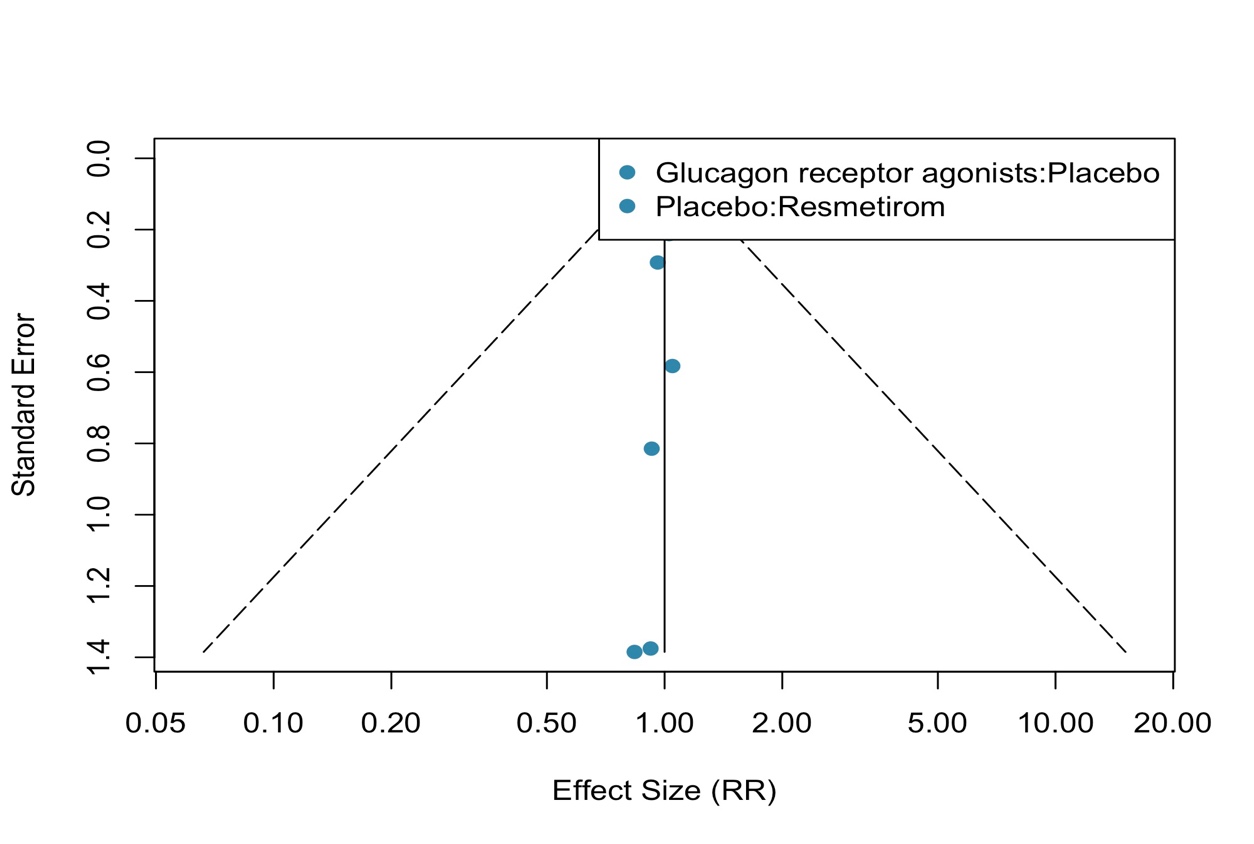


## Figure S6.10: Enhanced Liver Fibrosis (ELF)


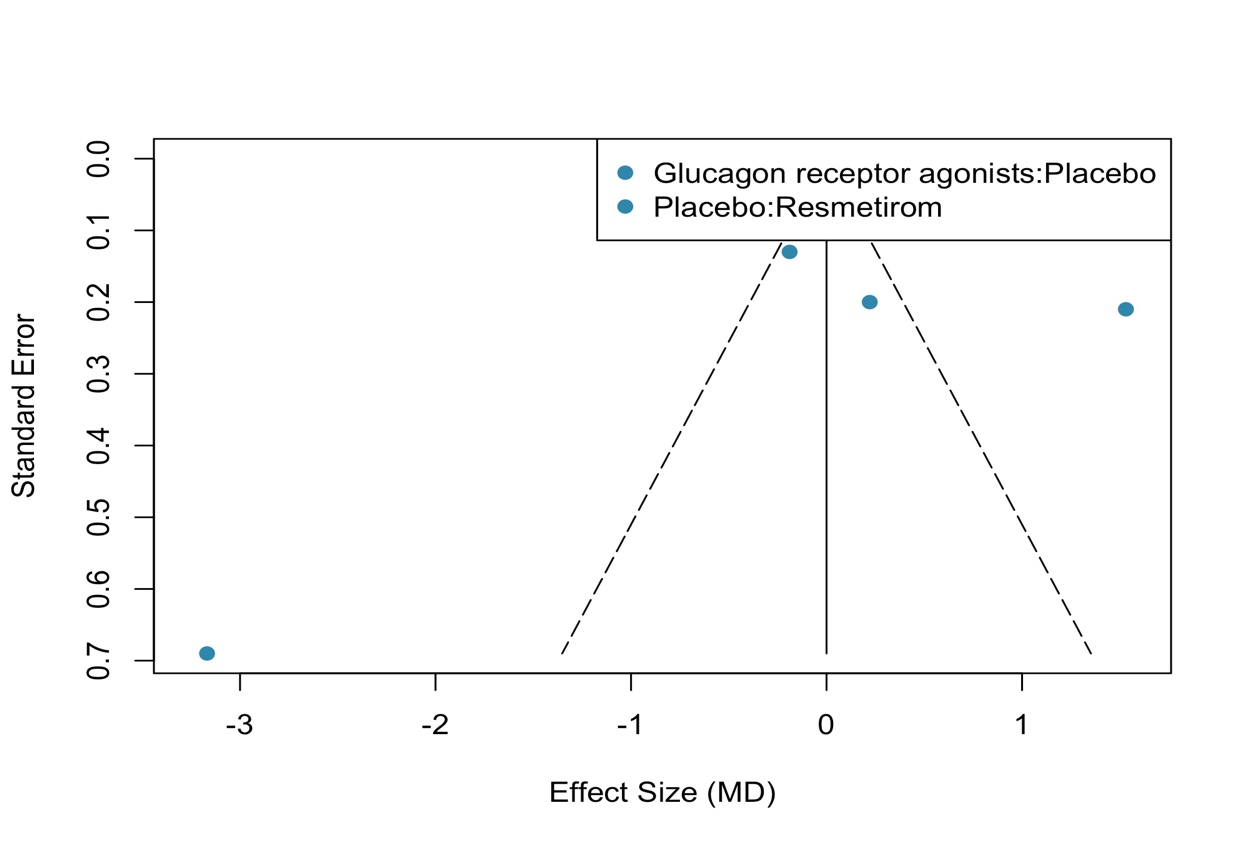


## Figure S6.11: Adiponectin


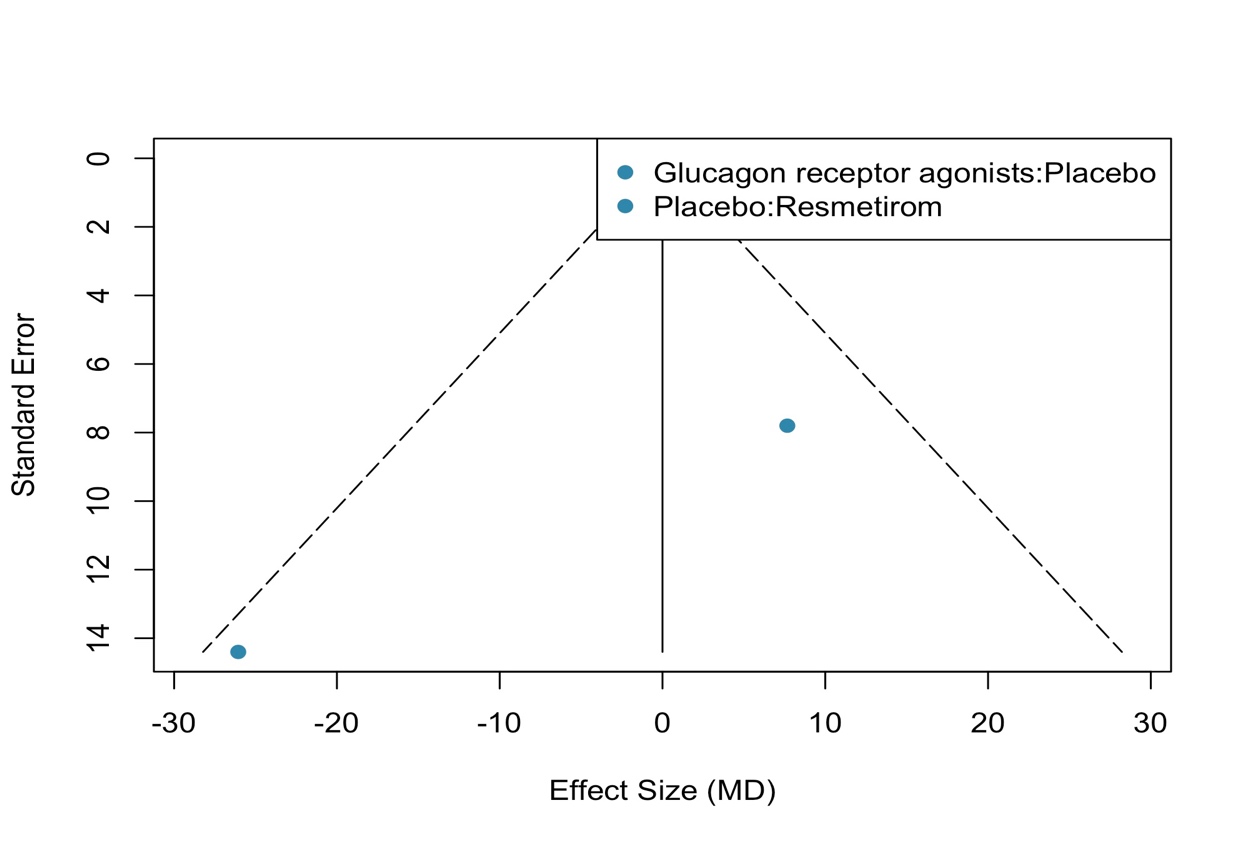


## Figure S6.12: MASH Resolution with No Worsening in Fibrosis


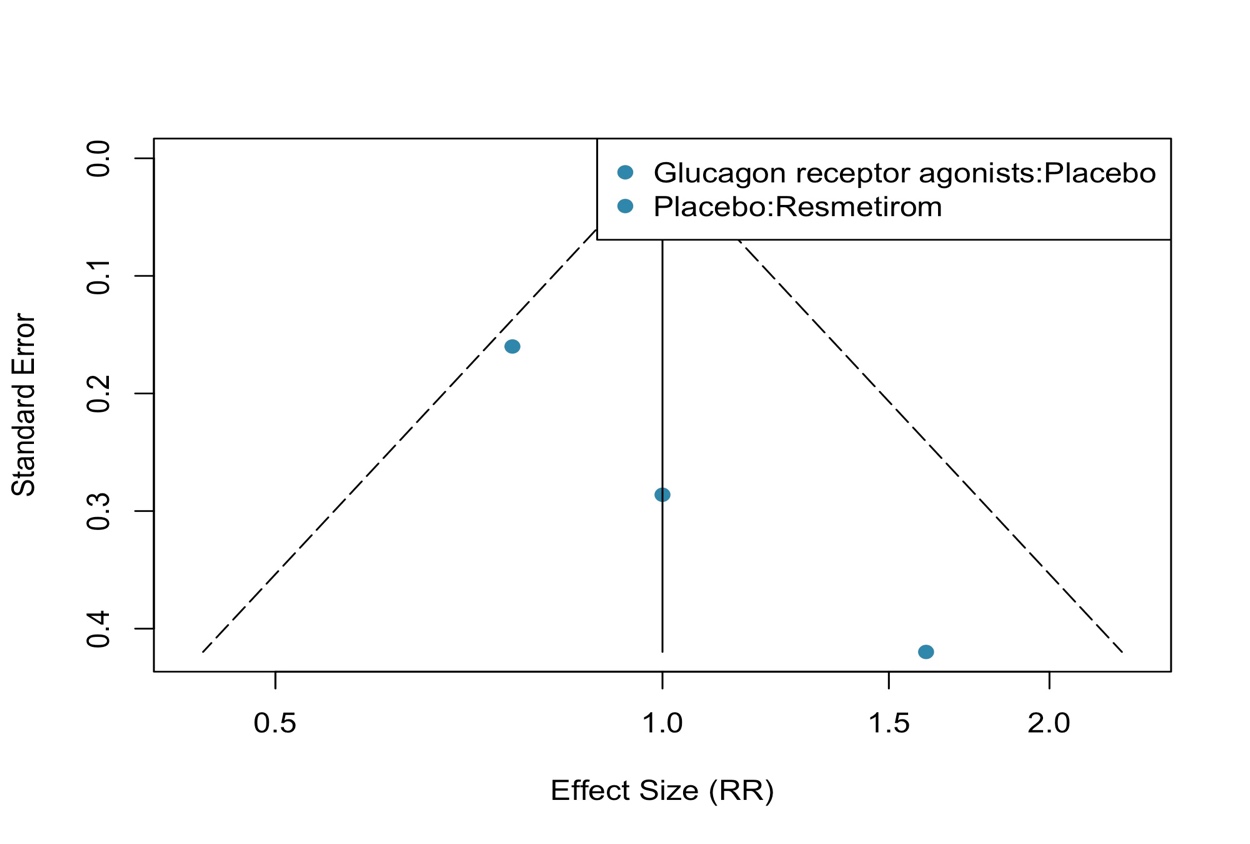


# **Supplement S7: League Tables**

## Table S7.1: ALT League

|  | Glucagon receptor agonists | Resmetirom | Placebo |
| --- | --- | --- | --- |
| Glucagon receptor agonists | Glucagon receptor agonists | . | -22.29 (-40.37; -4.22) |
| Resmetirom | -5.21 (-23.88; 13.47) | Resmetirom | -17.09 (-21.77; -12.40) |
| Placebo | -22.29 (-40.37; -4.22) | -17.09 (-21.77; -12.40) | Placebo |

## Table S7.2: AST League

|  | Glucagon receptor agonists | Resmetirom | Placebo |
| --- | --- | --- | --- |
| Glucagon receptor agonists | Glucagon receptor agonists | . | -17.45 (-32.59; -2.31) |
| Resmetirom | -7.65 (-23.16; 7.87) | Resmetirom | -9.80 (-13.19; -6.42) |
| Placebo | -17.45 (-32.59; -2.31) | -9.80 (-13.19; -6.42) | Placebo |

## Table S7.3: LDL League

|  | Resmetirom | Glucagon receptor agonists | Placebo |
| --- | --- | --- | --- |
| Resmetirom | Resmetirom | . | -17.34 (-20.54; -14.13) |
| Glucagon receptor agonists | -17.03 (-22.07; -11.99) | Glucagon receptor agonists | -0.31 ( -4.20; 3.58) |
| Placebo | -17.34 (-20.54; -14.13) | -0.31 ( -4.20; 3.58) | Placebo |

## Table S7.4: HDL League

|  | Resmetirom | Placebo | Glucagon receptor agonists |
| --- | --- | --- | --- |
| Resmetirom | Resmetirom | 2.11 ( 0.04; 4.19) | . |
| Placebo | 2.11 ( 0.04; 4.19) | Placebo | 0.19 (-0.72; 1.09) |
| Glucagon receptor agonists | 2.30 ( 0.04; 4.57) | 0.19 (-0.72; 1.09) | Glucagon receptor agonists |

## Table S7.5: Hepatic Fat Reduction (MRI-PDFF) League

|  | Glucagon receptor agonists | Resmetirom | Placebo |
| --- | --- | --- | --- |
| Glucagon receptor agonists | Glucagon receptor agonists | . | -12.12 (-15.42; -8.82) |
| Resmetirom | -4.34 ( -7.89; -0.78) | Resmetirom | -7.79 ( -9.10; -6.47) |
| Placebo | -12.12 (-15.42; -8.82) | -7.79 ( -9.10; -6.47) | Placebo |

## Table S7.6: Diarrhea League

|  | Glucagon receptor agonists | Resmetirom | Placebo |
| --- | --- | --- | --- |
| Glucagon receptor agonists | Glucagon receptor agonists | . | 2.30 (1.46; 3.61) |
| Resmetirom | 1.02 (0.62; 1.68) | Resmetirom | 2.25 (1.82; 2.79) |
| Placebo | 2.30 (1.46; 3.61) | 2.25 (1.82; 2.79) | Placebo |

## Table S7.7: Nausea League

|  | Glucagon receptor agonists | Placebo | Resmetirom |
| --- | --- | --- | --- |
| Glucagon receptor agonists | Placebo | 0.54 (0.41; 0.71) | 0.30 (0.20; 0.45) |
| Placebo | 0.54 (0.41; 0.71) | Resmetirom | . |
| Resmetirom | 0.30 (0.20; 0.45) | 0.55 (0.34; 0.90) | Glucagon receptor agonists |

## Table S7.8: Fatigue League

|  | Resmetirom | Placebo | Glucagon receptor agonists |
| --- | --- | --- | --- |
| Resmetirom | Resmetirom | 0.93 (0.62; 1.38) | . |
| Placebo | 0.93 (0.62; 1.38) | Placebo | 0.47 (0.20; 1.12) |
| Glucagon receptor agonists | 0.44 (0.17; 1.13) | 0.47 (0.20; 1.12) | Glucagon receptor agonists |

## Table S7.9: Serious Adverse Events League

|  | Resmetirom | Glucagon receptor agonists | Placebo |
| --- | --- | --- | --- |
| Resmetirom | Resmetirom | . | 1.13 (0.81; 1.57) |
| Glucagon receptor agonists | 0.99 (0.35; 2.78) | Glucagon receptor agonists | 1.15 (0.43; 3.06) |
| Placebo | 1.13 (0.81; 1.57) | 1.15 (0.43; 3.06) | Placebo |

## Table S7.10: Enhanced Liver Fibrosis (ELF) League

|  | Glucagon receptor agonists | Placebo | Resmetirom |
| --- | --- | --- | --- |
| Glucagon receptor agonists | Glucagon receptor agonists | -1.73 [-2.75; -0.71] | . |
| Placebo | -1.73 [-2.75; -0.71] | Placebo | 0.26 [-0.44; 0.96] |
| Resmetirom | -1.47 [-2.71; -0.23] | 0.26 [-0.44; 0.96] | Resmetirom |

## Table S7.11: Adiponectin League

|  | Glucagon receptor agonists | Placebo | Resmetirom |
| --- | --- | --- | --- |
| Glucagon receptor agonists | Glucagon receptor agonists | 32.73 [ 19.27; 46.20] | . |
| Placebo | 32.73 [ 19.27; 46.20] | Placebo | -0.95 [ -1.57; -0.32] |
| Resmetirom | 31.79 [ 18.31; 45.27] | -0.95 [ -1.57; -0.32] | Resmetirom |

## Table S7.12: MASH Resolution with No Worsening in Fibrosis League

|  | Glucagon receptor agonists | Placebo | Resmetirom |
| --- | --- | --- | --- |
| Glucagon receptor agonists | Glucagon receptor agonists | 2.423 [0.900; 6.524] | . |
| Placebo | 2.423 [0.900; 6.524] | Placebo | 0.490 [0.244; 0.984] |
| Resmetirom | 1.186 [0.353; 3.985] | 0.490 [0.244; 0.984] | Resmetirom |

# **Supplement S8: Treatment ranking for each outcome**

## Table S8.1: Change in ALT

| Rank | Intervention | P-Score |
| --- | --- | --- |
| First | Glucagon receptor agonists | 0.7746 |
| Second | Resmetirom | 0.7157 |
| Third | Placebo | 0.0098 |

## Table S8.2: Change in AST

| Rank | Intervention | P-Score |
| --- | --- | --- |
| First | Glucagon receptor agonists | 0.8030 |
| Second | Resmetirom | 0.6687 |
| Third | Placebo | 0.0283 |

## Table S8.3: Change in LDL

| Rank | Intervention | P-Score |
| --- | --- | --- |
| First | Resmetirom | 1.0000 |
| Second | Glucagon receptor agonists | 0.2812 |
| Third | Placebo | 0.2188 |

## Table S8.4: Change in HDL

| Rank | Intervention | P-Score |
| --- | --- | --- |
| First | Resmetirom | 0.9770 |
| Second | Placebo | 0.3402 |
| Third | Glucagon receptor agonists | 0.1828 |

## Table S8.5: Change in Hepatic Fat Fraction (MRI-PDFF)

| Rank | Intervention | P-Score |
| --- | --- | --- |
| First | Glucagon receptor agonists | 0.9130 |
| Second | Resmetirom | 0.5634 |
| Third | Placebo | 0.0236 |

## Table S8.6: Diarrhea

| Rank | Intervention | P-Score |
| --- | --- | --- |
| First | Glucagon receptor agonists | 0.7652 |
| Second | Resmetirom | 0.7348 |
| Third | Placebo | 0.0001 |

## Table S8.7: Nausea

| Rank | Intervention | P-Score |
| --- | --- | --- |
| First | Placebo | 0.9999 |
| Second | Resmetirom | 0.4894 |
| Third | Glucagon receptor agonists | 0.0107 |

## Table S8.8: Fatigue

| Rank | Intervention | P-Score |
| --- | --- | --- |
| First | Resmetirom | 0.8001 |
| Second | Placebo | 0.6556 |
| Third | Glucagon receptor agonists | 0.0443 |

## Table S8.9: Serious Adverse Events

| Rank | Intervention | P-Score |
| --- | --- | --- |
| First | Resmetirom | 0.6289 |
| Second | Glucagon receptor agonists | 0.5586 |
| Third | Placebo | 0.3125 |

## Table S8.10: Enhanced Liver Fibrosis (ELF)

| Rank | Intervention | P-Score |
| --- | --- | --- |
| First | Glucagon receptor agonists | 0.9948 |
| Second | Resmetirom | 0.3881 |
| Third | Placebo | 0.1171 |

## Table S8.11: Adiponectin

| Rank | Intervention | P-Score |
| --- | --- | --- |
| First | Glucagon receptor agonists | 1.0000 |
| Second | Resmetirom | 0.4992 |
| Third | Placebo | 0.0008 |

## Table S8.12: MASH Resolution with No Worsening of Fibrosis

| Rank | Intervention | P-Score |
| --- | --- | --- |
| First | Glucagon receptor agonists | 0.7844 |
| Second | Resmetirom | 0.6844 |
| Third | Placebo | 0.0312 |

# **Supplement S9. Certainty of evidence for each outcome**

## Table S9.1: Change in ALT

| Comparison | Within Study  Bias | Reporting Bias | Indirectness | Imprecision | Heterogeneity | Incoherence | Overall Confidence | Reasonf for Downgrade |
| --- | --- | --- | --- | --- | --- | --- | --- | --- |
| Placebo:Resmetirom | Some concerns | Some concerns | No concerns | Some concerns | Major concerns | No concerns | Low | Within-study bias,  Reporting bias,  Imprecision,  Heterogeneity |
| Glucagon receptor agonists:Placebo | Some concerns | Some concerns | No concerns | Some concerns | Major concerns | No concerns | Low | Within-study bias,  Reporting bias,  Imprecision,  Heterogeneity |
| Glucagon receptor agonists:Resmetirom | Some concerns | Some concerns | No concerns | Major concerns | Major concerns | No concerns | Very low | Within-study bias,  Reporting bias,  Imprecision,  Heterogeneity |

## Table S9.2: Change in AST

| Comparison | Within Study Bias | Reporting Bias | Indirectness | Imprecision | Heterogeneity | Incoherence | Overall Confidence | Reason for downgrade |
| --- | --- | --- | --- | --- | --- | --- | --- | --- |
| Placebo:Resmetirom | Some concerns | Some concerns | No concerns | Some concerns | Major concerns | No concerns | Low | Within-study bias, Reporting bias, Imprecision, Heterogeneity |
| Glucagon receptor agonists:Placebo | Some concerns | Some concerns | No concerns | Major concerns | Major concerns | No concerns | Very low | Within-study bias, Reporting bias, Imprecision, Heterogeneity |
| Glucagon receptor agonists:Resmetirom | Some concerns | Some concerns | No concerns | Major concerns | Major concerns | No concerns | Very low | Within-study bias, Reporting bias, Imprecision, Heterogeneity |

## Table S9.3: Change in LDL

| Comparison | Within Study Bias | Reporting Bias | Indirectness | Imprecision | Heterogeneity | Incoherence | Overall Confidence | Reason for Downgrade |
| --- | --- | --- | --- | --- | --- | --- | --- | --- |
| Placebo:Resmetirom | Some concerns | Some concerns | No concerns | Some concerns | No concerns | No concerns | Low | Within-study bias, Reporting bias, Imprecision |
| Glucagon receptor agonists:Placebo | Some concerns | Some concerns | No concerns | Major concerns | No concerns | No concerns | Low | Within-study bias, Reporting bias, Imprecision |
| Glucagon receptor agonists:Resmetirom | Some concerns | Some concerns | No concerns | Some concerns | No concerns | No concerns | Low | Within-study bias, Reporting bias, Imprecision |

## Table S9.4: Change in HDL

| Comparison | Within Study Bias | Reporting Bias | Indirectness | Imprecision | Heterogeneity | Incoherence | Overall  Confidence | Reason for Downgrade |
| --- | --- | --- | --- | --- | --- | --- | --- | --- |
| Placebo:Resmetirom | Some concerns | Some concerns | No concerns | Some concerns | No concerns | No concerns | Low | Within-study bias, Reporting bias, Imprecision |
| Glucagon receptor agonists:Placebo | Some concerns | Some concerns | No concerns | Major concerns | No concerns | No concerns | Low | Within-study bias, Reporting bias, Imprecision |
| Glucagon receptor agonists:Resmetirom | Some concerns | Some concerns | No concerns | Some concerns | No concerns | No concerns | Low | Within-study bias, Reporting bias, Imprecision |

## Table S9.5: Change in Hepatic Fat Fraction (MRI-PDFF)

| Comparison | Within Study Bias | Reporting Bias | Indirectness | Imprecision | Heterogeneity | Incoherence | Overall Confidence | Reason for Downgrade |
| --- | --- | --- | --- | --- | --- | --- | --- | --- |
| Placebo:Resmetirom | Some concerns | Some concerns | No concerns | Major concerns | Major concerns | No concerns | Very low | Within-study bias, Reporting bias, Imprecision, Heterogeneity |
| Glucagon receptor agonists:Placebo | Some concerns | Some concerns | No concerns | Some concerns | Major concerns | No concerns | Low | Within-study bias, Reporting bias, Imprecision, Heterogeneity |
| Glucagon receptor agonists:Resmetirom | Some concerns | Some concerns | No concerns | Major concerns | Major concerns | No concerns | Very low | Within-study bias, Reporting bias, Imprecision, Heterogeneity |

## Table S9.6: Diarrhea

| Comparison | Within Study Bias | Reporting Bias | Indirectness | Imprecision | Heterogeneity | Incoherence | Overall Confidence | Reason for Downgrade |
| --- | --- | --- | --- | --- | --- | --- | --- | --- |
| Placebo:Resmetirom | No concerns | Some concerns | Some concerns | Major concerns | No concerns | No concerns | Low | Reporting bias, Indirectness, Imprecision |
| Glucagon receptor agonists:Placebo | No concerns | Some concerns | Some concerns | No concerns | No concerns | No concerns | Moderate | Reporting bias, Indirectness |
| Glucagon receptor agonists:Resmetirom | No concerns | Some concerns | Some concerns | No concerns | No concerns | No concerns | Moderate | Reporting bias, Indirectness |

## Table S9.7: Nausea

| Comparison | Within Study Bias | Reporting Bias | Indirectness | Imprecision | Heterogeneity | Incoherence | Overall Confidence | Reason for Downgrade |
| --- | --- | --- | --- | --- | --- | --- | --- | --- |
| Placebo:Resmetirom | No concerns | Some concerns | Some concerns | No concerns | No concerns | No concerns | Moderate | Reporting bias, Indirectness |
| Glucagon receptor agonists:Placebo | No concerns | Some concerns | Some concerns | No concerns | No concerns | No concerns | Moderate | Reporting bias, Indirectness |
| Glucagon receptor agonists:Resmetirom | No concerns | Some concerns | Some concerns | No concerns | No concerns | No concerns | Moderate | Reporting bias, Indirectness |

## Table S9.8: Fatigue

| Comparison | Within Study Bias | Reporting Bias | Indirectness | Imprecision | Heterogeneity | Incoherence | Overall Confidence | Reason for Downgrade |
| --- | --- | --- | --- | --- | --- | --- | --- | --- |
| Placebo:Resmetirom | No concerns | Some concerns | Some concerns | No concerns | No concerns | No concerns | Moderate | Reporting bias, Indirectness |
| Glucagon receptor agonists:Placebo | No concerns | Some concerns | Some concerns | No concerns | No concerns | No concerns | Moderate | Reporting bias, Indirectness |
| Glucagon receptor agonists:Resmetirom | No concerns | Some concerns | Some concerns | No concerns | No concerns | No concerns | Moderate | Reporting bias, Indirectness |

## Table S9.9: Serious Adverse Events

| Comparison | Within Study Bias | Reporting Bias | Indirectness | Imprecision | Heterogeneity | Incoherence | Overall Confidence | Reason for Downgrade |
| --- | --- | --- | --- | --- | --- | --- | --- | --- |
| Placebo:Resmetirom | No concerns | Some concerns | Some concerns | No concerns | No concerns | No concerns | Moderate | Reporting bias, Indirectness |
| Glucagon receptor agonists:Placebo | No concerns | Some concerns | Some concerns | No concerns | No concerns | No concerns | Moderate | Reporting bias, Indirectness |
| Glucagon receptor agonists:Resmetirom | No concerns | Some concerns | Some concerns | Major concerns | No concerns | No concerns | Low | Reporting bias, Indirectness, Imprecision |

## Table S9.10: Enhanced Liver Fibrosis (ELF)

| Comparison | Within Study Bias | Reporting Bias | Indirectness | Imprecision | Heterogeneity | Incoherence | Overall Confidence | Reason for Downgrade |
| --- | --- | --- | --- | --- | --- | --- | --- | --- |
| Placebo:Resmetirom | Some concerns | Some concerns | No concerns | Major concerns | Major concerns | No concerns | Very low | Within-study bias, Reporting bias, Imprecision, Heterogeneity |
| Glucagon receptor agonists:Placebo | Some concerns | Some concerns | No concerns | Some concerns | Major concerns | No concerns | Low | Within-study bias, Reporting bias, Imprecision, Heterogeneity |
| Glucagon receptor agonists:Resmetirom | Some concerns | Some concerns | No concerns | Some concerns | Major concerns | No concerns | Low | Within-study bias, Reporting bias, Imprecision, Heterogeneity |

## Table S9.11: Adiponectin

| Comparison | Within Study Bias | Reporting Bias | Indirectness | Imprecision | Heterogeneity | Incoherence | Overall Confidence | Reason for Downgrade |
| --- | --- | --- | --- | --- | --- | --- | --- | --- |
| Placebo:Resmetirom | Some concerns | Some concerns | No concerns | No concerns | Major concerns | No concerns | Low | Within-study bias, Reporting bias, Heterogeneity |
| Glucagon receptor agonists:Placebo | Some concerns | Some concerns | No concerns | Some concerns | Major concerns | No concerns | Low | Within-study bias, Reporting bias, Imprecision, Heterogeneity |
| Glucagon receptor agonists:Resmetirom | Some concerns | Some concerns | No concerns | Some concerns | Major concerns | No concerns | Low | Within-study bias, Reporting bias, Imprecision, Heterogeneity |

## Table S9.12: MASH Resolution with No Worsening of Fibrosis

| Comparison | Within Study Bias | Reporting Bias | Indirectness | Imprecision | Heterogeneity | Incoherence | Overall Confidence | Reason for Downgrade |
| --- | --- | --- | --- | --- | --- | --- | --- | --- |
| Placebo:Resmetirom | No concerns | Some concerns | Some concerns | Major concerns | Major concerns | No concerns | Very low | Reporting bias, Indirectness, Imprecision, Heterogeneity |
| Glucagon receptor agonists:Placebo | No concerns | Some concerns | Some concerns | No concerns | Major concerns | No concerns | Low | Reporting bias, Indirectness, Heterogeneity |
| Glucagon receptor agonists:Resmetirom | No concerns | Some concerns | Some concerns | Major concerns | Major concerns | No concerns | Very low | Reporting bias, Indirectness, Imprecision, Heterogeneity |

# **Supplement S10. Sensitivity Analysis**

## Table S10.1: Leave-One-Out-Analysis for ALT

| **Study Omitted** | **I^2^** | **Tau^2^** | **Interpretation** |
| --- | --- | --- | --- |
| Harrison,2019 | 67.8% | 79.2717 | Effect Stable |
| Harrison,2023 | 0% | 0 | Effect Stable |
| Harrison,2024 | 15.3% | 17.3297 | Effect Stable |
| Shankar,2024 | 71.3% | 78.1669 | Effect Stable |
| Sanyal_a,2024 | 70.5% | 75.2869 | Effect Stable |
| Sanyal_b,2024 | 71.6% | 76.9097 | Effect Stable |

## Table S10.2: Leave-One-Out-Analysis for AST

| **Study Omitted** | **I^2^** | **Tau^2^** | **Interpretation** |
| --- | --- | --- | --- |
| Harrison,2019 | 85.2% | 182.1472 | Effect Stable |
| Harrison,2023 | 58.8% | 57.3760 | Effect Stable |
| Harrison,2024 | 0% | 0 | Effect Stable |
| Shankar,2024 | 85.3% | 88.4147 | Effect Stable |
| Sanyal_a,2024 | 85.3% | 88.4649 | Effect Stable |
| Sanyal_b,2024 | 85.3% | 87.4851 | Effect Stable |

## Table S10.3: Leave-One-Out Analysis for LDL

| **Study Omitted** | **I^2^** | **Tau^2^** | **Interpretation** |
| --- | --- | --- | --- |
| Harrison,2019 | 26.8% | 4.6376 | Effect Stable |
| Harrison,2023 | 0% | 0 | Effect Stable |
| Harrison,2024 | 0% | 0 | Effect Stable |
| Shankar,2024 | 85.3% | 88.4147 | Effect Stable |
| Sanyal_b,2024 | 26.8% | 4.7450 | Effect Stable |

## Table S10.4: Leave-One-Out Analysis for HDL

| **Study Omitted** | **I^2^** | **Tau^2^** | **Interpretation** |
| --- | --- | --- | --- |
| Harrison,2019 | 0% | 0 | Effect Stable |
| Harrison,2023 | 0% | 0 | Effect Stable |
| Harrison,2024 | 0% | 0 | Effect Stable |
| Shankar,2024 | 0% | 0 | Effect Stable |
| Sanyal_b,2024 | 0% | 0 | Effect Stable |

## Table S10.5: Leave-One-Out Analysis for Change in Hepatic Fat Fraction (MRI-PDFF)

| **Study Omitted** | **I^2^** | **Tau^2^** | **Interpretation** |
| --- | --- | --- | --- |
| Harrison,2019 | 97.7% | 763.1010 | Effect Stable |
| Harrison,2023 | 98.7% | 853.4641 | Effect Stable |
| Harrison,2024 | 98.5% | 812.1313 | Effect Stable |
| Shankar,2024 | 98.1% | 450.1900 | Effect Stable |
| Sanyal_a,2024 | 98.7% | 593.8251 | Effect Stable |
| Sanyal_b,2024 | 98.6% | 585.8855 | Effect Stable |

## Table S20.6: Leave-One-Out Analysis for Diarrhea

| **Study Omitted** | **I^2^** | **Tau^2^** | **Interpretation** |
| --- | --- | --- | --- |
| Harrison,2019 | 0% | 0 | Effect Stable |
| Harrison,2023 | 0% | 0 | Effect Stable |
| Harrison,2024 | 0% | 0 | Effect Stable |
| Shankar,2024 | 0% | 0 | Effect Stable |
| Sanyal_a,2024 | 0% | 0 | Effect Stable |
| Sanyal_b,2024 | 0% | 0 | Effect Stable |

## Table S10.7: Leave-One-Out Analysis for Nausea

| **Study Omitted** | **I^2^** | **Tau^2^** | **Interpretation** |
| --- | --- | --- | --- |
| Harrison,2019 | 33.7% | 0.0498 | Effect Stable |
| Harrison,2023 | 10.5% | 0.0284 | Effect Stable |
| Harrison,2024 | 0% | 0 | Effect Stable |
| Shankar,2024 | 11.7% | 0.0136 | Effect Stable |
| Sanyal_a,2024 | 31.5% | 0.0418 | Effect Stable |
| Sanyal_b,2024 | 0% | 0 | Effect Stable |

## Table S10.8: Leave-One-Out Analysis for Fatigue

| **Study Omitted** | **I^2^** | **Tau^2^** | **Interpretation** |
| --- | --- | --- | --- |
| Harrison,2019 | 0% | 0 | Effect Stable |
| Harrison,2023 | 0% | 0 | Effect Stable |
| Harrison,2024 | 0% | 0 | Effect Stable |
| Sanyal_a,2024 | 0% | 0 | Effect Stable |
| Sanyal_b,2024 | 0% | 0 | Effect Stable |

## Table S10.9: Leave-On-Out Analysis for Serious Adverse Events

| **Study Omitted** | **I^2^** | **Tau^2^** | **Interpretation** |
| --- | --- | --- | --- |
| Harrison,2019 | 0% | 0 | Effect Stable |
| Harrison,2023 | 0% | 0 | Effect Stable |
| Harrison,2024 | 0% | 0 | Effect Stable |
| Shankar,2024 | 0% | 0 | Effect Stable |
| Sanyal_a,2024 | 0% | 0 | Effect Stable |
| Sanyal_b,2024 | 0% | 0 | Effect Stable |

## Table S10.10: Leave-On-Out Analysis for Enhanced Liver Fibrosis (ELF)

| **Study Omitted** | **I^2^** | **Tau^2^** | **Interpretation** |
| --- | --- | --- | --- |
| Harrison,2019 | 95.4% | 0.4964 | Effect Stable |
| Harrison,2023 | 95.4% | 0.9172 | Effect Stable |
| Harrison,2024 | 95.6% | 1.1135 | Effect Stable |
| Shankar,2024 | 34.9% | 0.0094 | Effect Stable |
| Sanyal_a,2024 | 34.9% | 0.0094 | Effect Stable |

## Table S10.11: Leave-On-Out Analysis for Adiponectin

| **Study Omitted** | **I^2^** | **Tau^2^** | **Interpretation** |
| --- | --- | --- | --- |
| Harrison,2019 | 82.1% | 0.4446 | Effect Stable |
| Harrison,2023 | 56.9% | 0.1749 | Effect Stable |
| Harrison,2024 | 70.9% | 0.3224 | Effect Stable |
| Shankar,2024 | 72.4% | 0.1520 | Effect Stable |
| Sanyal_a,2024 | 72.4% | 0.1520 | Effect Stable |

## Table S10.12: Leave-On-Out Analysis for MASH Resolution with No Worsening of Fibrosis

| **Study Omitted** | **I^2^** | **Tau^2^** | **Interpretation** |
| --- | --- | --- | --- |
| Harrison,2019 | NA | NA | NA |
| Harrison,2024 | NA | NA | NA |
| Sanyal_b,2024 | 63.2% | 0.1735 | Effect Stable |

# **Supplement S11. PRISMA checklist**

| Section and Topic | Item # | Checklist item | Location where item is reported |
| --- | --- | --- | --- |
| TITLE | | |  |
| Title | 1 | Identify the report as a systematic review. | 1 |
| ABSTRACT | | |  |
| Abstract | 2 | See the PRISMA 2020 for Abstracts checklist. | 3 |
| INTRODUCTION | | |  |
| Rationale | 3 | Describe the rationale for the review in the context of existing knowledge. | 4 |
| Objectives | 4 | Provide an explicit statement of the objective(s) or question(s) the review addresses. | 4 |
| METHODS | | |  |
| Eligibility criteria | 5 | Specify the inclusion and exclusion criteria for the review and how studies were grouped for the syntheses. | 5 |
| Information sources | 6 | Specify all databases, registers, websites, organisations, reference lists and other sources searched or consulted to identify studies. Specify the date when each source was last searched or consulted. | 6 |
| Search strategy | 7 | Present the full search strategies for all databases, registers and websites, including any filters and limits used. | 6 |
| Selection process | 8 | Specify the methods used to decide whether a study met the inclusion criteria of the review, including how many reviewers screened each record and each report retrieved, whether they worked independently, and if applicable, details of automation tools used in the process. | 7 |
| Data collection process | 9 | Specify the methods used to collect data from reports, including how many reviewers collected data from each report, whether they worked independently, any processes for obtaining or confirming data from study investigators, and if applicable, details of automation tools used in the process. | 7 |
| Data items | 10a | List and define all outcomes for which data were sought. Specify whether all results that were compatible with each outcome domain in each study were sought (e.g. for all measures, time points, analyses), and if not, the methods used to decide which results to collect. | 7 |
|  | 10b | List and define all other variables for which data were sought (e.g. participant and intervention characteristics, funding sources). Describe any assumptions made about any missing or unclear information. | 7 |
| Study risk of bias assessment | 11 | Specify the methods used to assess risk of bias in the included studies, including details of the tool(s) used, how many reviewers assessed each study and whether they worked independently, and if applicable, details of automation tools used in the process. | 7 |
| Effect measures | 12 | Specify for each outcome the effect measure(s) (e.g. risk ratio, mean difference) used in the synthesis or presentation of results. | 7 |
| Synthesis methods | 13a | Describe the processes used to decide which studies were eligible for each synthesis (e.g. tabulating the study intervention characteristics and comparing against the planned groups for each synthesis (item #5)). | 8-9 |
|  | 13b | Describe any methods required to prepare the data for presentation or synthesis, such as handling of missing summary statistics, or data conversions. | 7 |
|  | 13c | Describe any methods used to tabulate or visually display results of individual studies and syntheses. | 6, 9 |
|  | 13d | Describe any methods used to synthesize results and provide a rationale for the choice(s). If meta-analysis was performed, describe the model(s), method(s) to identify the presence and extent of statistical heterogeneity, and software package(s) used. | 7 |
|  | 13e | Describe any methods used to explore possible causes of heterogeneity among study results (e.g. subgroup analysis, meta-regression). | 7 |
|  | 13f | Describe any sensitivity analyses conducted to assess robustness of the synthesized results. | 7-8 |
| Reporting bias assessment | 14 | Describe any methods used to assess risk of bias due to missing results in a synthesis (arising from reporting biases). | 7 |
| Certainty assessment | 15 | Describe any methods used to assess certainty (or confidence) in the body of evidence for an outcome. | 7 |
| RESULTS | | |  |
| Study selection | 16a | Describe the results of the search and selection process, from the number of records identified in the search to the number of studies included in the review, ideally using a flow diagram. | 9 |
|  | 16b | Cite studies that might appear to meet the inclusion criteria, but which were excluded, and explain why they were excluded. | 9 |
| Study characteristics | 17 | Cite each included study and present its characteristics. | 8 |
| Risk of bias in studies | 18 | Present assessments of risk of bias for each included study. | - |
| Results of individual studies | 19 | For all outcomes, present, for each study: (a) summary statistics for each group (where appropriate) and (b) an effect estimate and its precision (e.g. confidence/credible interval), ideally using structured tables or plots. | 10-16 |
| Results of syntheses | 20a | For each synthesis, briefly summarise the characteristics and risk of bias among contributing studies. | 10-16 |
|  | 20b | Present results of all statistical syntheses conducted. If meta-analysis was done, present for each the summary estimate and its precision (e.g. confidence/credible interval) and measures of statistical heterogeneity. If comparing groups, describe the direction of the effect. | 10-16 |
|  | 20c | Present results of all investigations of possible causes of heterogeneity among study results. | 10-16 |
|  | 20d | Present results of all sensitivity analyses conducted to assess the robustness of the synthesized results. | 10-16 |
| Reporting biases | 21 | Present assessments of risk of bias due to missing results (arising from reporting biases) for each synthesis assessed. | 10-16 |
| Certainty of evidence | 22 | Present assessments of certainty (or confidence) in the body of evidence for each outcome assessed. | 10-16 |
| DISCUSSION | | |  |
| Discussion | 23a | Provide a general interpretation of the results in the context of other evidence. | 18-20 |
|  | 23b | Discuss any limitations of the evidence included in the review. | 18-20 |
|  | 23c | Discuss any limitations of the review processes used. | 18-20 |
|  | 23d | Discuss implications of the results for practice, policy, and future research. | 18-20 |
| OTHER INFORMATION | | |  |
| Registration and protocol | 24a | Provide registration information for the review, including register name and registration number, or state that the review was not registered. | 5 |
|  | 24b | Indicate where the review protocol can be accessed, or state that a protocol was not prepared. | 5 |
|  | 24c | Describe and explain any amendments to information provided at registration or in the protocol. | 5 |
| Support | 25 | Describe sources of financial or non-financial support for the review, and the role of the funders or sponsors in the review. | 2 |
| Competing interests | 26 | Declare any competing interests of review authors. | 2 |
| Availability of data, code and other materials | 27 | Report which of the following are publicly available and where they can be found: template data collection forms; data extracted from included studies; data used for all analyses; analytic code; any other materials used in the review. | - |
